# *IRX1* suppresses cervical squamous cell carcinoma progression by downregulating *ACLY* expression to inhibit lipid synthesis

**DOI:** 10.1016/j.isci.2026.117115

**Published:** 2026-09-01

**Authors:** Xiangyu Liu, Xinwei Hou, Yuanyuan Zhang, Jindong Sheng, Yue Yu, Xuchen Cao, Ke Wang

**Affiliations:** 1 Department of Gynaecological Oncology, Tianjin Medical University Cancer Institute and Hospital, National Clinical Research Center for Cancer, Key Laboratory of Cancer Prevention and Therapy, Tianjin, Tianjin’s Clinical Research Center for Cancer, Tianjin 300060, China; 2 The First Department of Breast Cancer, Tianjin Medical University Cancer Institute and Hospital, National Clinical Research Center for Cancer, Key Laboratory of Cancer Prevention and Therapy, Tianjin, Tianjin’s Clinical Research Center for Cancer, Tianjin 300060, China; 3 Department of Oncology, Haihe Hospital, Tianjin University, Tianjin, China

**Keywords:** ACLY, cervical squamous cell carcinoma, IRX1, lipid synthesis, NME3

## Abstract

*IRX1* functions as a tumor suppressor in cervical cancer; however, its underlying mechanism remains unclear. In this study, we observed that *IRX1* expression is frequently downregulated in cervical cancer cells owing to promoter hypermethylation. Functionally, *IRX1* overexpression suppressed cell proliferation, migration, and invasion, whereas *IRX1* knockdown enhanced these malignant phenotypes. Mechanistically, *IRX1* directly bound to the ATP-citrate lyase (*ACLY*) promoter and recruited *NME3*, thereby forming a transcriptional repressive complex. This *IRX1*-*NME3*/*ACLY* axis inhibited *de novo* fatty acid synthesis, as reflected by reduced levels of acetyl-CoA, malonyl-CoA, triglycerides, cholesterol, and lipid droplets. Consistently, *ACLY* overexpression partially reversed the suppressive effects of *IRX1* on lipid metabolism and tumor progression. Collectively, our findings reveal that *IRX1* functions as a metabolic tumor suppressor by transcriptionally repressing *ACLY*-mediated lipogenesis.

## Highlights

• IRX1 is downregulated in cervical cancer due to promoter methylation
• IRX1 suppresses lipid synthesis by transcriptionally repressing ACLY expression
• IRX1 recruits NME3 to form a transcriptional repressive complex on the ACLY promoter
• The IRX1-NME3/ACLY axis represents a potential metabolic target for cervical cancer

## Graphical abstract

**Figure:**
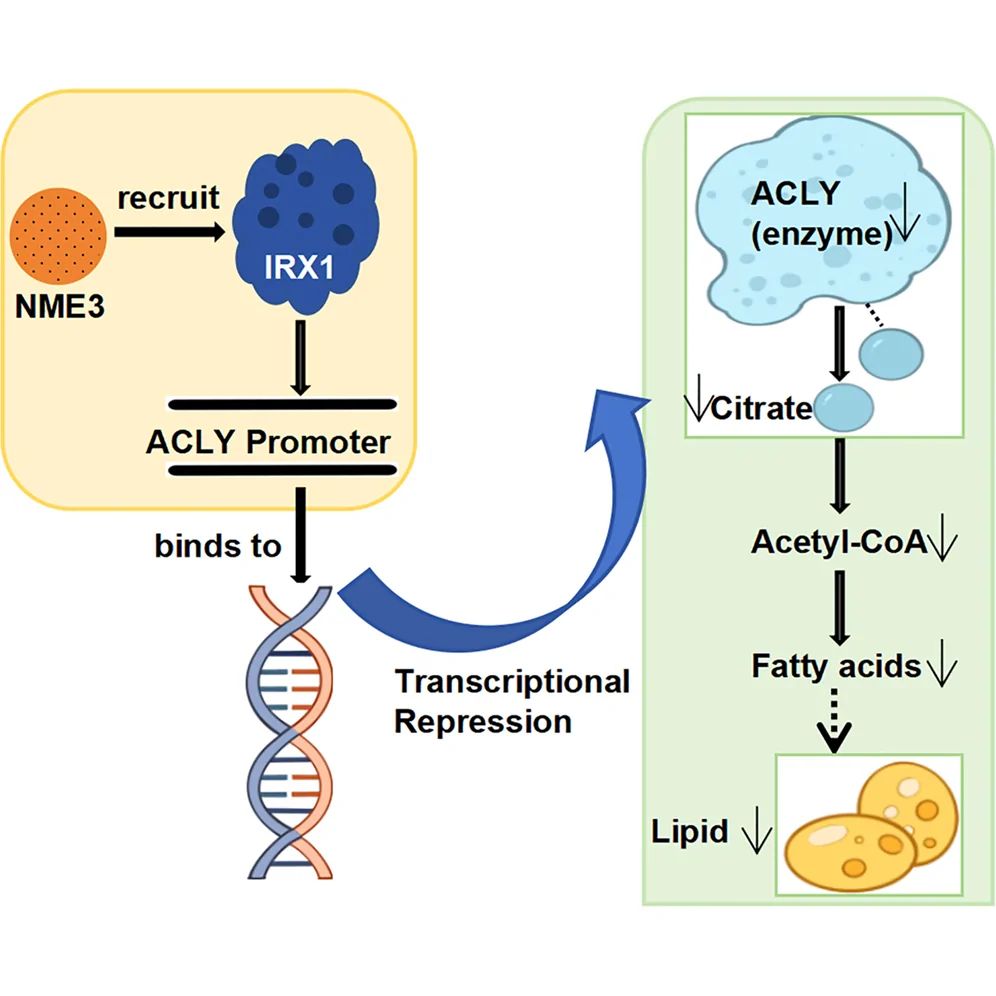

## Teaser

Biological sciences; Molecular biology; Cell biology

## Introduction

Cervical cancer is a malignant tumor of the cervix, primarily caused by persistent infection with high-risk types of the human papillomavirus (HPV). Early-stage cervical cancer is often asymptomatic, underscoring the critical importance of regular screening via Pap smears and HPV testing for secondary prevention.^1^^,^^2^^,^^3^ For advanced cervical cancer, targeted therapies and immunotherapies have emerged as promising treatment avenues, though they remain predominantly within the realm of clinical investigation.^4^ Future efforts should focus on enhancing HPV vaccine coverage, improving the accessibility and quality of screening, and developing therapeutic agents to alleviate the global burden of this disease.^5^^,^^6^

*IRX1*, a member of the Iroquois family, encodes a transcription factor crucial for embryonic development.^7^^,^^8^ Beyond its developmental functions, *IRX1* has been implicated as a key regulator in the development and progression of multiple cancer types. In gastric cancer, for instance, *IRX1* expression is frequently downregulated via promoter hypermethylation, an alteration correlated with advanced tumor stage and poor prognosis.^9^ Supporting this, Guo et al. demonstrated that *IRX1* silencing in gastric cancer cells is mediated by promoter hypermethylation, and that its re-expression suppresses cell proliferation and invasion.^10^ Similarly, in non-small cell lung cancer (NSCLC), Küster et al. reported that *IRX1* promoter methylation is a significant prognostic factor, with low *IRX1* expression correlating with reduced patient survival, suggesting its potential as a diagnostic biomarker.^11^ Furthermore, in head and neck squamous cell carcinoma (HNSCC), Bennett et al. Identified *IRX1* promoter methylation as an event closely associated with tumor progression.^12^ Collectively, these studies support the notion of *IRX1* as a tumor suppressor across multiple cancer types. However, its regulatory mechanisms and therapeutic potential in cervical squamous cell carcinoma (CESC) remain to be fully elucidated.

Our study demonstrates that *IRX1* functions as a tumor suppressor in CESC by transcriptionally repressing ATP-citrate lyase (*ACLY*), thereby inhibiting *de novo* fatty acid synthesis and curbing malignant progression. Furthermore, we identified nucleoside diphosphate kinase 3 (*NME3*) as an essential transcriptional co-repressor that cooperates with *IRX1* to silence *ACLY* expression. Taken together, our results define an *IRX1*-*NME3*/*ACLY* regulatory axis that plays a pivotal role in driving CESC progression, revealing a previously unrecognized mechanism linking transcriptional regulation to metabolic reprogramming.

## Results

### *IRX1* inhibits migration and proliferation of CESC cells

**Figure 1:**
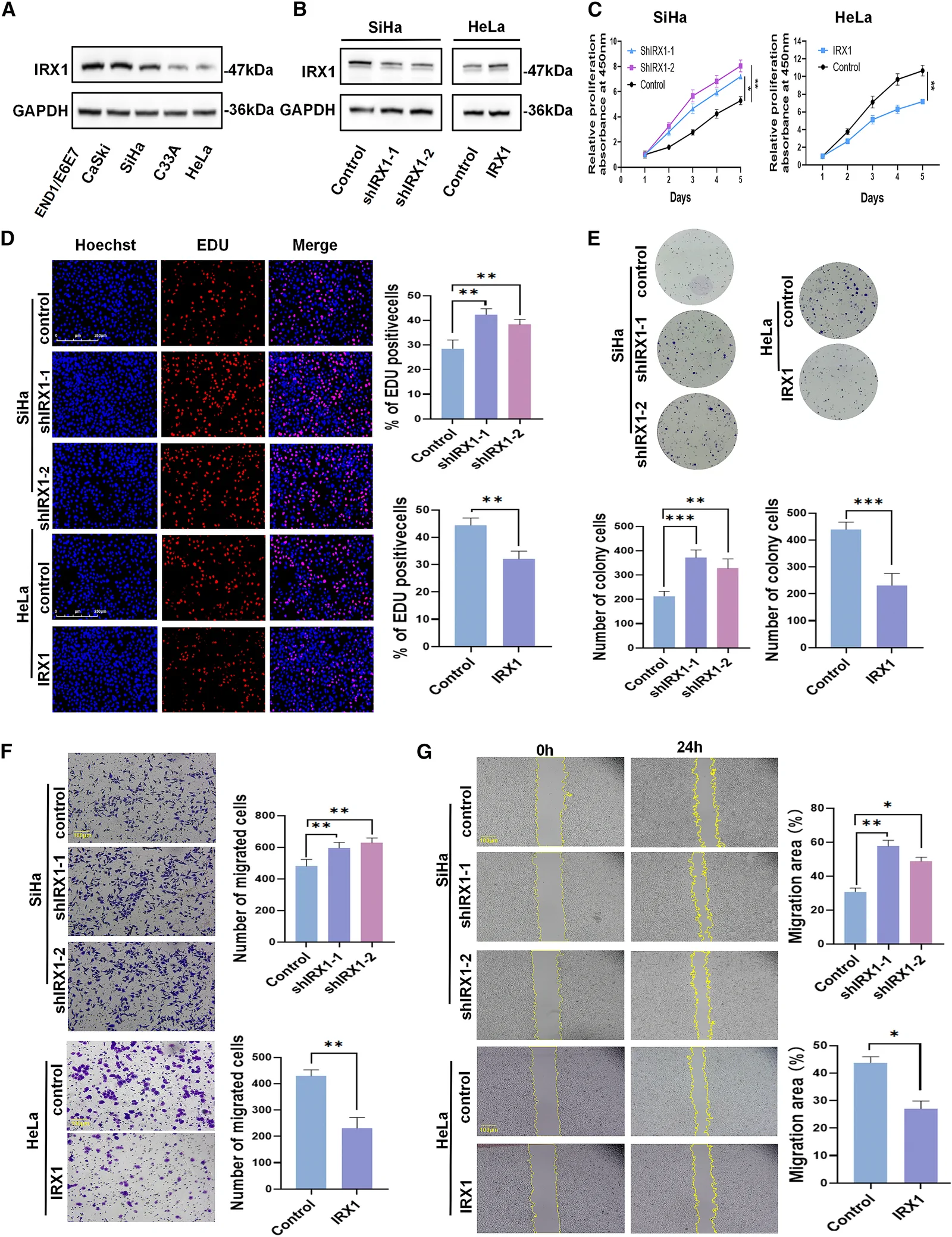
*IRX1* inhibits CESC proliferation and migration *in vitro* See also Figure S1. (A) The IRX1 expression in five CESC cell lines (End1/E6E7, HeLa, C33A, SiHa, CaSki) by western blot. (B) The IRX1 expression in *IRX1*-knockdown SiHa cells (left) or *IRX1*-overexpressing HeLa cells (right), as well as control cells, by western blot. (C–E) The effect of *IRX1* on CESC proliferation was determined by CCK-8 (C), EdU (D), and colony formation (E) assays. Scale bars, 250 μm (D). (F and G) The effect of *IRX1* on CESC migration and invasion was determined by Transwell (F) and wound healing (G) assays. Scale bars, 100 μm. Data are presented as mean ± SD from three independent biological replicates (*n* = 3) ∗*p* < 0.05, ∗∗*p* < 0.01, ∗∗∗*p* < 0.001 by unpaired two-tailed *t* test (C (right), D (down), E (right), F (down), G (down)) and one-way ANOVA followed by Tukey’s post hoc test (C (left), D (up), E (left), F (up), G (up)).

Western blot analysis revealed differential expression of IRX1 protein across cell lines (Figure 1A). IRX1 levels were lower in cervical cancer cell lines (HeLa, C33A, SiHa, CaSki) compared to the non-tumorigenic cervical epithelial cell line End1/E6E7. Notably, IRX1 levels were relatively lower in HeLa and C33A cells but higher in SiHa and CaSki cells. To investigate the functional role of *IRX1*, we established stable *IRX1*-overexpressing HeLa and C33A cell lines, as well as *IRX1*-knockdown SiHa and CaSki cell lines. Successful modulation of IRX1 expression was confirmed by western blotting (Figure 1B; Figure S1A). The impact of *IRX1* on cell proliferation was assessed using functional assays. CCK-8, EdU, and colony formation assays demonstrated that *IRX1* overexpression resulted in a lower proliferation rate, a reduced percentage of EdU-positive cells, and decreased colony-forming capacity compared to control cells (Figures 1C–1E). Conversely, *IRX1* knockdown enhanced all these proliferative capabilities (Figures S1B–S1D). Transwell and wound healing assays showed that migratory ability was inhibited in *IRX1*-overexpressing cells (Figures 1F and 1G) but enhanced in *IRX1*-knockdown cells (Figures S1E and S1F). Collectively, these results indicate that *IRX1* functions as a tumor suppressor in CESC.

### Promoter DNA methylation epigenetically modulates *IRX1* expression

**Figure 2:**
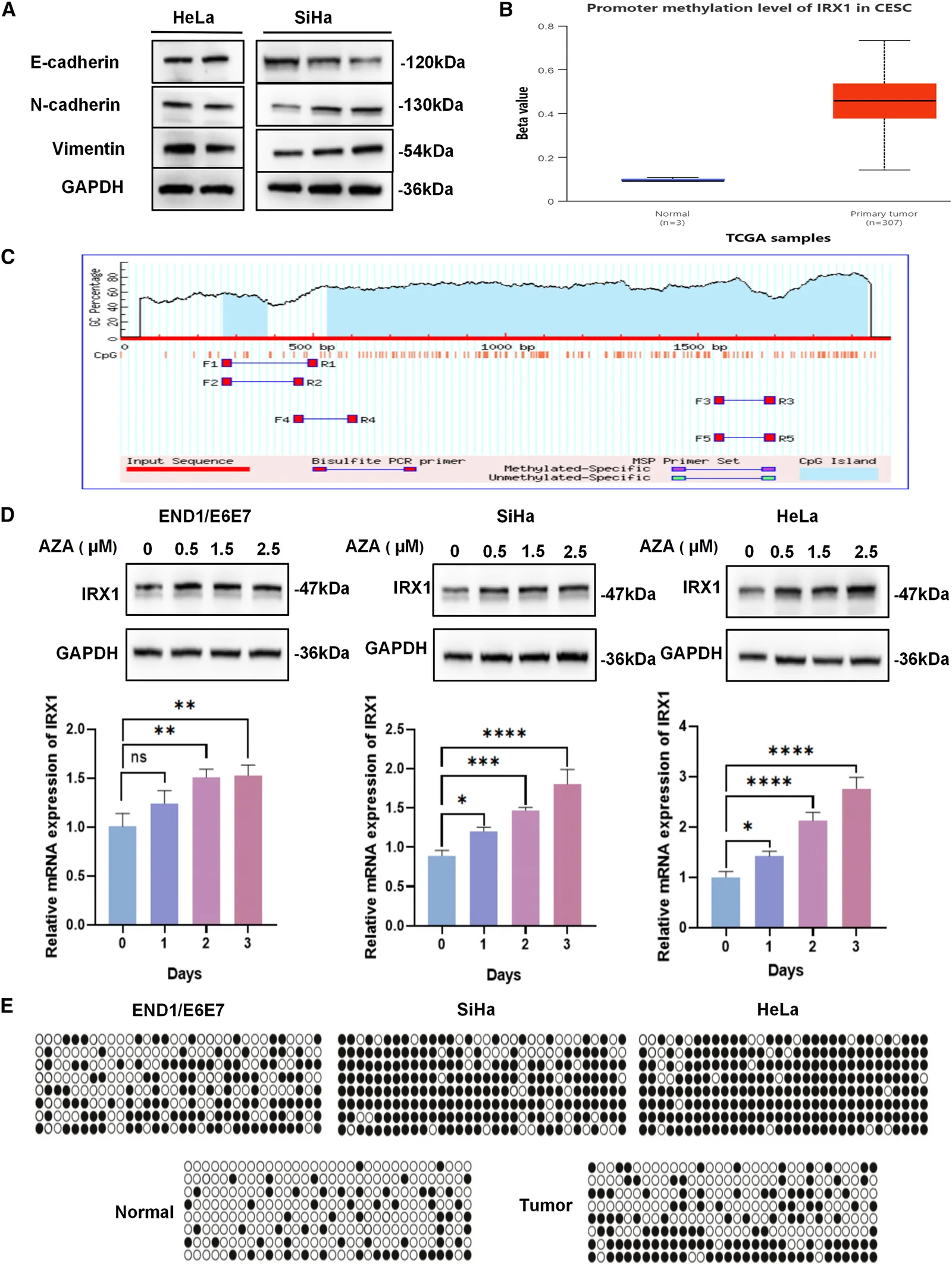
Promoter DNA methylation epigenetically modulates *IRX1* expression (A) The expression of EMT markers (E-cadherin, Vimentin, and N-cadherin) in *IRX1*-overexpressing HeLa cells (left) or *IRX1*-knockdown SiHa cells (right), as well as control cells, by western blot. (B) The *IRX1* promoter methylation levels of normal and tumor tissues in CESC by the UALCAN database (TCGA, normal tissues, *n* = 3; primary tumor tissues, *n* = 307). (C) The CpG island on the *IRX1* promoter region predicted using MethPrimer software. (D) The *IRX1* expression after treatment of AZA in a dose-escalation manner (0.5 μm, 1.5 μm and 2.5 μm) in END1/E6E7 cells or HeLa cells, as well as SiHa cells, by western blotting (up) and RT-qPCR (down). (E) The promoter methylation status of *IRX1* in three CESC cells (End1/E6E7, HeLa, SiHa) and one paired tissue detected by BSP. Data are presented as mean ± SD from three independent biological replicates (*n* = 3). ∗*p* < 0.05, ∗∗*p* < 0.01, and ∗∗∗*p* < 0.001 by one-way ANOVA followed by Tukey’s post hoc test (D).

Promoter hypermethylation is a key epigenetic mechanism that silences tumor suppressor genes. Consistent with this paradigm, bioinformatic analysis and experimental evidence indicated that *IRX1* expression is negatively regulated by promoter methylation in cervical cancer. Having established *IRX1*’s tumor-suppressive function, we next examined its effect on the expression of epithelial-mesenchymal transition (EMT)-associated markers. Western blot analysis of EMT markers revealed that *IRX1*-overexpressing HeLa cells exhibited increased E-cadherin and decreased N-cadherin and vimentin levels. Conversely, *IRX1*-knockdown SiHa cells showed the opposite pattern (Figure 2A). Given that lipid metabolism is known to regulate EMT, these changes in EMT-associated markers are consistent with the role of *IRX1* in suppressing migration and invasion. We then analyzed the *IRX1* promoter methylation status in public databases. Data from UALCAN (https://ualcan.path.uab.edu/analysis.html) showed higher *β*-values in cervical cancer tissues compared to normal cervical tissues (Figure 2B). *In silico* analysis using MethPrimer (http://www.urogene.org/methprimer) identified the CpG island spanning the *IRX1* promoter region (Figure 2C), providing a potential target for methylation-mediated regulation. To functionally validate this epigenetic regulation, we treated End1/E6E7, HeLa, and SiHa cells with the DNA methyltransferase inhibitor 5-aza-2′-deoxycytidine (AZA) at concentrations of 0.5, 1.5, and 2.5 μm. After 96 h of treatment, RT-qPCR and western blot analyses showed that AZA treatment dose-dependently restored *IRX1* expression in the cancer cell lines (HeLa and SiHa), which display high basal promoter methylation. In contrast, only a marginal increase was observed in the low-methylation End1/E6E7 cells (Figure 2D). Finally, bisulfite sequencing PCR (BSP) was employed to directly assess methylation at the nucleotide level. This analysis confirmed higher methylation densities at the *IRX1* promoter CpG island in cervical cancer cell lines and clinical tissue samples relative to their normal counterparts (Figure 2E). Together, these data establish that promoter hypermethylation is a primary mechanism for the transcriptional silencing of the tumor suppressor *IRX1* in cervical cancer.

### *IRX1* inhibits lipid metabolism in cervical cancer cells

**Figure 3:**
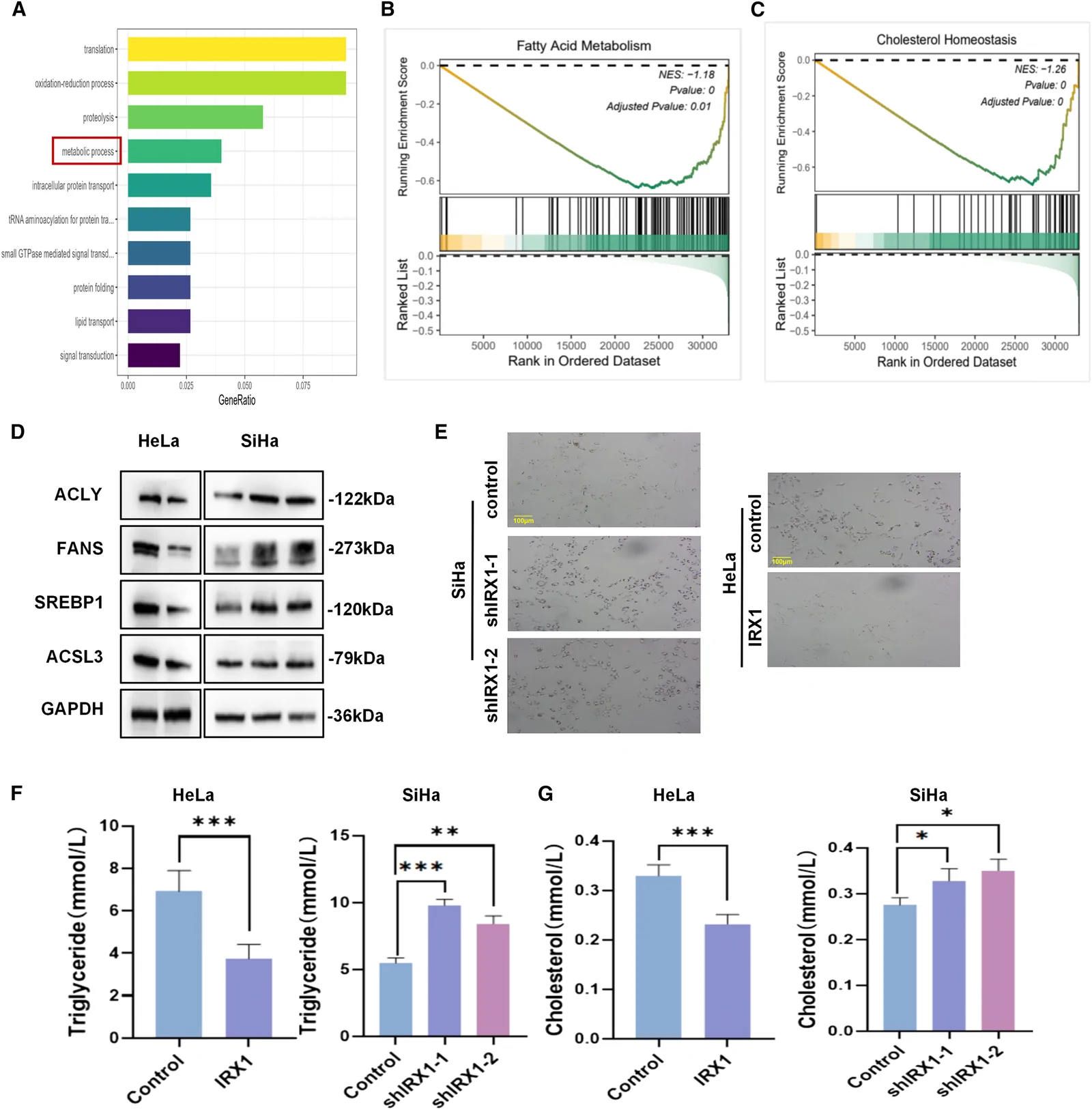
*IRX1* inhibits lipid metabolism in CESC cells See also Figure S2. (A) The top 10 enriched terms of differentially expressed genes detected by GO. (B and C) GSEA of differentially expressed genes. (D) The expression of lipid metabolism-related genes (ACLY, FANS, SREBP1, ACSL3) by western blot. (E) The lipid droplet abundance in *IRX1*-overexpressing HeLa cells or *IRX1*-knockdown SiHa cells, as well as control cells, detected by Oil Red O staining. Scale bars, 100 μm. (F and G) The measurement of triglyceride (F) and cholesterol (G) contents in *IRX1*-overexpressing HeLa cells or *IRX1*-knockdown SiHa, as well as control cells. Data are presented as mean ± SD from three independent biological replicates (*n* = 3). ∗*p* < 0.05, ∗∗*p* < 0.01, and ∗∗∗*p* < 0.001 by unpaired two-tailed *t* test (F (left), G (left)) and one-way ANOVA followed by Tukey’s post hoc test (F (right), G (right)).

To elucidate the molecular basis of *IRX1* function in cervical cancer, RNA-seq analysis was performed on control and *IRX1*-overexpressing HeLa cells. Gene Ontology (GO:http://www.gsea-msigdb.org/gsea) indicated enrichment in metabolic pathways (Figure 3A). Gene set enrichment analysis (GSEA: http://www.gsea-msigdb.org/gsea) showed that the differentially expressed genes were significantly enriched in pathways related to lipid metabolism and cholesterol metabolism (Figures 3B and 3C), suggesting a potential role for *IRX1* in these processes. Western blot analysis of key lipid metabolism enzymes showed that *IRX1* overexpression reduced the protein levels of ACSL3, SREBP1, FASN, and ACLY, whereas *IRX1* knockdown increased their levels (Figure 3D; Figure S2A). These results suggest that *IRX1* suppresses *de novo* fatty acid synthesis by downregulating key enzymes in this pathway, identifying *ACLY* as a critical downstream target. To further validate the role of *IRX1* in lipid metabolism, Oil Red O staining revealed a significant decrease in lipid droplet abundance in *IRX1*-overexpressing cells (Figure 3E) and an increase in *IRX1*-knockdown cells (Figure S2B), providing visual confirmation that *IRX1* inhibits lipid storage. Furthermore, measurement of key metabolites showed that levels of triglycerides (Figure 3F; Figure S2C) and cholesterol (Figure 3G; Figure S2D) were decreased upon *IRX1* overexpression and increased upon its knockdown. These results further substantiate the role of *IRX1* in inhibiting lipid synthesis and metabolic flux. In summary, these results consistently demonstrate that *IRX1* acts as an effective inhibitor of lipid metabolism in CESC, operating through the downregulation of key metabolic enzymes and the consequent reduction in metabolite accumulation.

### *IRX1* regulates lipid synthesis by exerting transcriptional repression on *ACLY* gene expression

**Figure 4:**
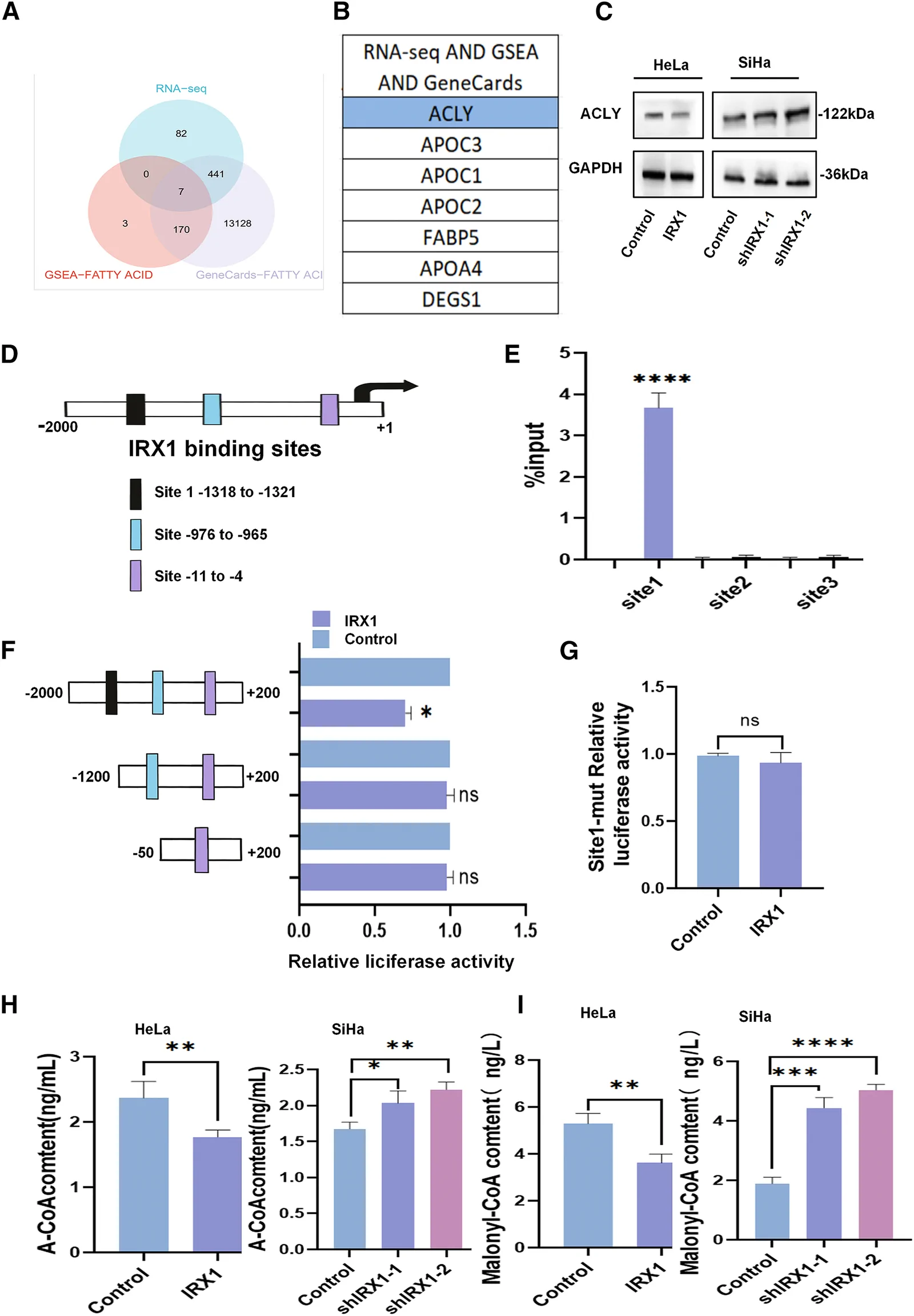
*IRX1* regulates lipid synthesis by exerting transcriptional repression on *ACLY* gene expression See also Figure S2. (A and B) Integration of RNA-seq data and lipid metabolism-related gene sets (GSEA and GeneCards). (C) The ACLY expression in *IRX1*-overexpressing HeLa cells or *IRX1*-knockdown SiHa cells, as well as the control cells, by western blot. (D) Three potential *IRX1* binding sites on the *ACLY* promoter predicted using the HumanTF database. (E) The interaction between *IRX1* and the *ACLY* promoter region was verified by ChIP analysis. (F and G) The effect of *IRX1* on *ACLY* promoter activity (F) or mutant *ACLY* promoter activity (G) was determined by dual-luciferase analysis. (H and I) The measurement of acetyl-CoA (H) and malonyl-CoA (I) contents in *IRX1*-overexpressing HeLa cells or *IRX1*-knockdown SiHa cells, as well as control cells. Data are presented as mean ± SD from three independent biological replicates (*n* = 3). ∗*p* < 0.05, ∗∗*p* < 0.01, and ∗∗∗*p* < 0.001 by unpaired two-tailed *t* test (F, G, H (left), I (left)) and one-way ANOVA followed by Tukey’s post hoc test (E, H (right), I (right)).

Next, we performed an integrated analysis to identify specific targets through which *IRX1* represses lipid metabolism. By cross-referencing RNA-seq data with GSEA and the GeneCards database, focusing on fatty acid metabolism gene sets, the results showed that seven overlapping genes were identified by a Venn diagram (Figures 4A and 4B). Among the seven overlapping genes identified by the Venn diagram, ACLY was selected for two reasons: it is located at the metabolic branchpoint of both fatty acid and cholesterol synthesis pathways enriched by IRX1, and it exhibited the largest differential expression in the RNA-seq data among all candidates. To determine if *ACLY* mediates the metabolic effects of *IRX1*, we manipulated ACLY expression in both *IRX1*-overexpressing and *IRX1*-knockdown cell lines. Western blot analysis confirmed the successful modulation of ACLY protein levels in these settings (Figure 4C). Using the HumanTF database (https://humantfs.ccbr.utoronto.ca/allTFs.php), we analyzed the *IRX1* binding motif and identified three potential binding sites within the *ACLY* promoter region (Figure 4D): (5′-TGTTATCT-3′) at positions -1328 to -1321, (5′-CAACATAGCAAA-3′) at positions -976 to -965, and (5′-AAAAAACA-3′) at positions -11 to -4. ChIP confirmed that *IRX1* specifically binds to site 1 of the *ACLY* promoter, located between positions -1328 and -1321 (Figure 4E). We constructed a series of truncated *ACLY* promoter-driven luciferase reporter vectors to map the functional binding region. Dual-luciferase reporter assays demonstrated that *IRX1* overexpression repressed the transcriptional activity of the promoter fragment containing site 1. Next, we constructed an *ACLY* promoter reporter with a mutated *IRX1* binding site 1 (5′-TGCGGCCT-3′). In contrast to the wild-type promoter reporter, *IRX1* failed to suppress the activity of this mutant reporter (Figures 4F and 4G). To further investigate the role of *IRX1* in lipid metabolism, we next measured the levels of key metabolites: acetyl-CoA and malonyl-CoA. *IRX1* overexpression markedly decreased acetyl-CoA and malonyl-CoA levels. Conversely, *IRX1* knockdown elevated these metabolite levels(Figures 4H and 4I; Figures S2E and S2F). In summary, these data indicate that *IRX1* regulates lipid metabolism by directly binding to the *ACLY* promoter and repressing its transcription.

### *IRX1* promotes nuclear translocation of NME3 to co-regulate *ACLY* expression

**Figure 5:**
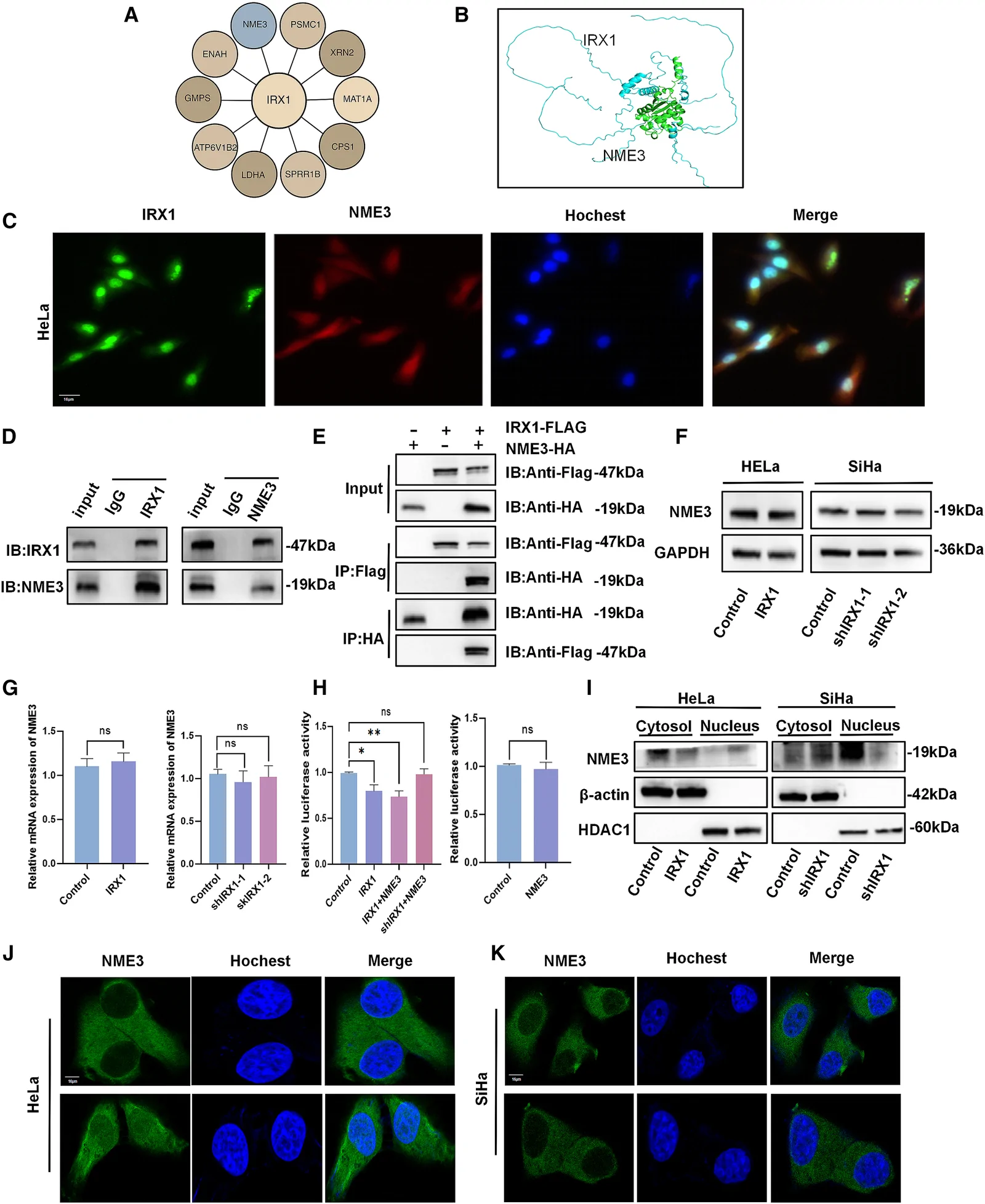
*IRX1* promotes nuclear translocation of NME3 to regulate *ACLY* expression (A) The top 10 *IRX1*-interacting genes identified by mass spectrometry. (B) The predictive 3D structure model of IRX1 and NME3. (C) The co-localization of IRX1 and NME3 in HeLa cells visualized by immunofluorescence (IF). Scale bars, 16 μm. (D and E) The interaction of IRX1 and NME3, including endogenous (D) and exogenous (E), by co-immunoprecipitation (CoIP). (F and G) The NME3 expression in *IRX1*-overexpressing HeLa cells or *IRX1*-knockdown SiHa cells, as well as control cells, by western blotting (F) and RT-qPCR (G). (H) The effect on NME3-dependent regulation of *ACLY* promoter activity determined by dual-luciferase analysis. (I–K) The NME3 expression in the nucleus and cytoplasm by western blotting (I) and immunofluorescence (J, K). Scale bars, 16 μm. Data are presented as mean ± SD from three independent biological replicates (*n* = 3). ∗*p* < 0.05, ∗∗*p* < 0.01, and ∗∗∗*p* < 0.001 by unpaired two-tailed *t* test (G (left), H (right)) and one-way ANOVA followed by Tukey’s post hoc test (G (right), H (left)).

Co-immunoprecipitation followed by mass spectrometry was performed to identify proteins interacting with IRX1. NME3 emerged as a high-confidence candidate (Figure 5A). We hypothesized that IRX1 interacts with NME3 to facilitate the transcriptional repression of *ACLY*. The interaction binding model between IRX1 and NME3 was predicted using the PDBePISA (https://www.ebi.ac.uk/pdbe/pisa/), GRAMM (https://gramm.compbio.ku.edu/), and PyMOL software (PyMOL molecular graphics system) packages (Figure 5B). Immunofluorescence assay was performed to detect the co-localization of IRX1 and NME3 in *IRX1* overexpressing HeLa cells; the result revealed significant nuclear co-localization of IRX1 and NME3 (Figure 5C). The interaction between IRX1 and NME3 was further verified by both endogenous (Figure 5D) and exogenous co-immunoprecipitation assays (Figure 5E), These results demonstrate that IRX1 interacts with NME3 in CESC cells. Subsequently, mRNA and protein expression levels of NME3 were determined by RT-qPCR and western blotting, respectively, in cells with either *IRX1* overexpression or knockdown. The results indicated that IRX1 did not affect NME3 expression (Figures 5F and 5G). Given the previously verified binding of *IRX1* to the *ACLY* promoter, we employed a dual-luciferase reporter assay to test whether *NME3* overexpression influences *IRX1*-mediated transcriptional regulation of *ACLY* (Figure 5H). The results demonstrated that *NME3* overexpression alone had no effect on *ACLY* promoter activity. However, co-expression of NME3 with IRX1 enhanced the transcriptional repression mediated by *IRX1* at *ACLY* site 1. Conversely, in cells with *IRX1* knockdown, *NME3* overexpression resulted in an attenuated repressive effect on the promoter. Collectively, these findings suggest that NME3 binds to IRX1 and likely may act through a recruitment mechanism. To further validate this hypothesis, nuclear-cytoplasmic fractionation and immunofluorescence analysis revealed that *IRX1* overexpression enhanced NME3 nuclear accumulation, whereas *IRX1* knockdown reduced its nuclear localization (Figures 5I and 5J). Collectively, these findings suggest that *IRX1* facilitates the nuclear recruitment of NME3, thereby assembling a transcriptional regulatory complex that co-regulates *ACLY* expression.

### *IRX1*-*NME3*/*ACLY* axis mediates fatty acid synthesis

**Figure 6:**
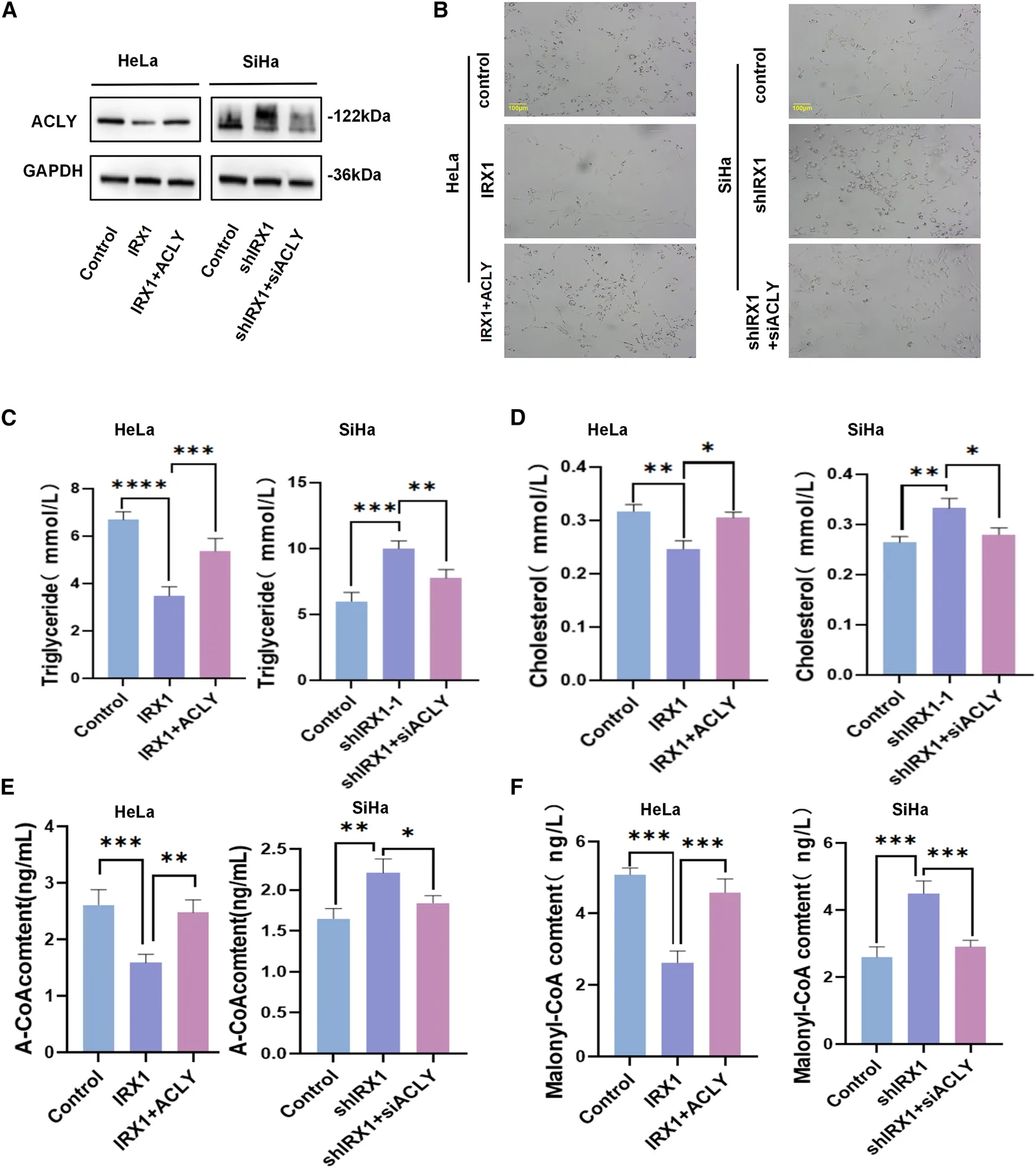
*IRX1*-*NME3*/*ACLY* axis mediates fatty acid synthesis (A) The ACLY expression in *IRX1*/*ACLY*-overexpressing HeLa cells or *IRX1*/*ACLY*-knockdown SiHa cells, as well as control cells by western blot. (B) The lipid droplet abundance in cells as in (A) was detected by Oil Red O staining. Scale bars, 100 μm. (C and D) The measurement of triglyceride (C) and cholesterol (D) contents in cells as in (A). (E and F) The measurement of Acetyl-CoA (E) and malonyl-CoA (F) levels by ELISA under the same conditions as in (A). Data are presented as mean ± SD from three independent biological replicates (*n* = 3). ∗*p* < 0.05, ∗∗*p* < 0.01, and ∗∗∗*p* < 0.001 by one-way ANOVA followed by Tukey’s post hoc test (C, D, E, F).

To investigate whether the IRX1/NME3 complex regulates *de novo* fatty acid synthesis via *ACLY*, we conducted rescue experiments in which *ACLY* was overexpressed in *IRX1*-overexpressing HeLa cells and knocked down in *IRX1*-knockdown SiHa cells. Western blot analysis showed that ACLY protein levels decreased upon *IRX1* overexpression, and this effect was reversed by subsequent *ACLY* overexpression. Conversely, ACLY levels increased upon *IRX1* knockdown, and this effect was similarly reversed by concomitant *ACLY* knockdown (Figure 6A). Consistent with the Western blot results, modulating ACLY expression reversed the effects of *IRX1* on cellular lipid accumulation, as measured by triglyceride and cholesterol levels, as well as by Oil Red O staining (Figures 6B–6D; Figures S2G and S2H). *ACLY* plays a critical role in lipid metabolism by catalyzing the cleavage of citrate to generate acetyl-CoA, a direct precursor for fatty acid synthesis. The acetyl-CoA generated serves as the substrate for malonyl-CoA production; together, these metabolites coordinate the progression of fatty acid synthesis and the maintenance of lipid metabolism. Acetyl-CoA and malonyl-CoA levels were quantified using commercial ELISA kits. In *IRX1*-overexpressing HeLa cells, subsequent *ACLY* overexpression reversed the reduction in acetyl-CoA and malonyl-CoA levels. Conversely, in *IRX1*-knockdown SiHa cells, additional *ACLY* knockdown reversed the elevation of these metabolites (Figures 6E and 6F; Figures S2I and S2K). These findings collectively indicate that the IRX1/NME3 complex regulates lipid metabolism by modulating *ACLY*.

### The *IRX1*-*NME3*/*ACLY* axis inhibits CESC proliferation and migration *in vitro*

**Figure 7:**
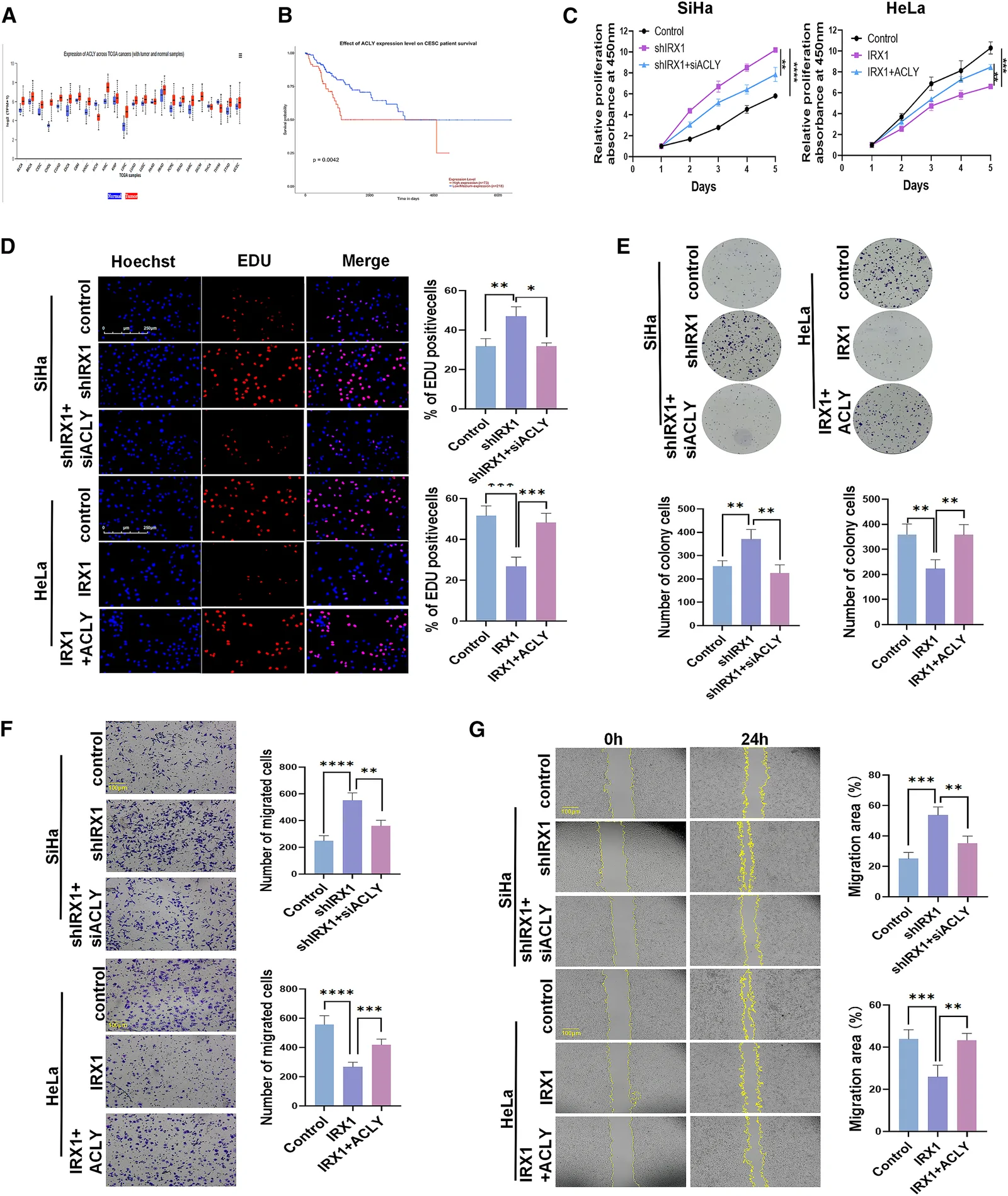
The *IRX1*-*NME3*/*ACLY* axis inhibits CESC proliferation and migration *in vitro* (A) The *ACLY* expression in multiple cancer types by the UALCAN database. (B) The survival analysis in patients with CESC with different *ACLY* expression levels predicted by the KM plotter. (C–E) The effect of *IRX1*-*NME3*/*ACLY* on CESC proliferation was determined by CCK-8 (C), EdU (D), and colony formation (E) assays. Scale bars, 250 μm (D). (F and G) The effect of *IRX1*-*NME3*/*ACLY* on CESC migration and invasion was determined by Transwell (F) and wound healing (G) assays. Scale bars, 100 μm. Data are presented as mean ± SD from three independent biological replicates (*n* = 3). ∗*p* < 0.05, ∗∗*p* < 0.01, and ∗∗∗*p* < 0.001 by one-way ANOVA followed by Tukey’s post hoc test (C, D, E, F, G).

*ACLY* expression was upregulated in CESC tissues compared to normal tissues from the UALCAN database (https://ualcan.path.uab.edu/analysis.html) (Figure 7A). Survival analysis indicated that elevated *ACLY* expression was associated with poorer overall prognosis in patients with CESC (Figure 7B). Using the established *IRX1*-overexpressing and *IRX1*-knockdown stable cell lines as backgrounds, we further generated corresponding *ACLY*-overexpressing and *ACLY*-knockdown derivatives. Functional assays including CCK-8, EdU, and colony formation demonstrated that modulating *ACLY* expression (either overexpression or knockdown) counteracted the effects of *IRX1* manipulation on cell proliferation (Figures 7C–7E). Similarly, Transwell migration and wound healing assays showed that altering *ACLY* expression also impacted the migratory capacity of these cells (Figures 7F and 7G). Collectively, these rescue experiments demonstrate that the IRX1-NME3 axis exerts its tumor-suppressive effects by transcriptionally repressing *ACLY*, thereby reducing proliferation, migration, and fatty acid synthesis in cervical cancer cells.

**Figure 8:**
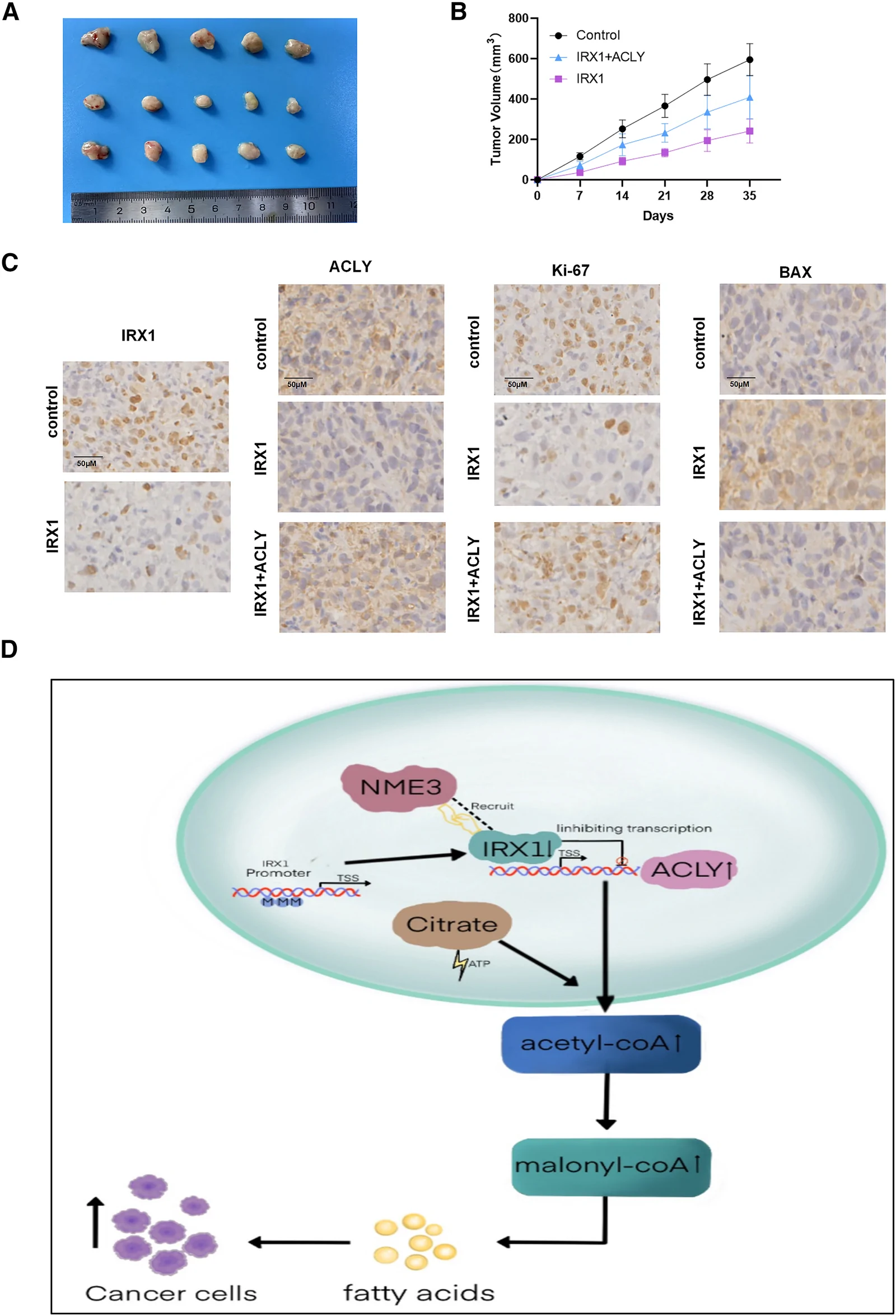
*IRX1* inhibits the progression of CESC by *ACLY in vivo* (A and B) The images (A) and tumor growth curves (B) of subcutaneous xenografts in NSG mice in *IRX1*-overexpressing HeLa cells or *IRX1*/*ACLY*co-overexpressing, as well as control cells. (*n* = 5). (C) The expression levels of IRX1, ACLY, Ki-67, and BAX by IHC. Scale bars, 50 μm. (D) The working model of the *IRX1*-*NME3*/*ACLY* axis in CESC progression. ∗*p* < 0.05, ∗∗*p* < 0.01, and ∗∗∗*p* < 0.001 by one-way ANOVA followed by Tukey’s post hoc test (B).

The tumor-suppressive role of *IRX1* was further validated *in vivo* using a xenograft mouse model. Subcutaneous injection of control HeLa cells, *IRX1*-overexpressing HeLa cells, or *IRX1*-overexpressing HeLa cells with *ACLY* overexpression into NOD-SCID γ (NSG) mice revealed that *IRX1* overexpression inhibited tumor growth compared to the control group. In contrast, concurrent overexpression of *ACLY* in the *IRX1*-overexpressing xenografts partially restored tumor growth (Figures 8A and 8B). These *in vivo* results confirm that *IRX1* acts as a tumor growth inhibitor in cervical cancer. This effect is likely mediated, at least in part, through the suppression of pro-proliferative signaling pathways. Immunohistochemical staining confirmed that *ACLY* expression was downregulated in *IRX1*-overexpressing tumors, but restored in the *IRX1*/*ACLY*-overexpressing tumor. Notably, the changes in proliferation and apoptosis-related markers induced by *IRX1* were abolished by concurrent *ACLY* overexpression (Figure 8C). In summary, our work delineates the *IRX1*-*NME3*/*ACLY* axis as a key regulatory node in cervical cancer.

## Discussion

Reprogramming of lipid metabolism is a well-established hallmark of cancer, as it provides essential building blocks for membrane biogenesis, energy storage, and synthesis of signaling molecules. Transcription factors play a central role in coordinating this metabolic adaptation through regulating the expression of key lipogenic enzymes. Therefore, identifying the upstream regulators of lipid metabolism is crucial for understanding tumor progression and for developing targeted therapies. Here, we demonstrate that the transcription factor *IRX1* recruits NME3 to form a functional complex that represses the expression of *ACLY*, a key enzyme in *de novo* lipogenesis. This inhibition of fatty acid synthesis contributes to the suppression of cervical cancer progression. Collectively, our findings define the *IRX1*-*NME3*/*ACLY* axis as a critical regulator of metabolic reprogramming in cervical cancer cells and provide new mechanistic insights into tumor suppression.

In this study, both HPV-positive (HeLa, SiHa, CaSki) and HPV-negative (C33A) cells were used. The key findings were consistently observed across all cell lines, indicating that the *IRX1-NME3/ACLY* axis operates independently of HPV status. While HPV-specific modulation may exist and warrants further investigation, it does not affect the core conclusions of this study.

The transcription factor Iroquois homeobox 1 (*IRX1*) is recognized as a tumor suppressor, and its promoter methylation status is closely associated with tumorigenesis and progression in multiple cancer types.^13^^,^^14^^,^^15^ In CESC, *IRX1* expression is frequently downregulated compared to normal cervical tissue, a phenomenon often attributed to promoter hypermethylation.

*NME3*, a member of the nucleoside diphosphate kinase (NDPK) family, is implicated in tumorigenesis and cancer progression.^16^ The expression level of *NME3* is a significant prognostic indicator across multiple cancer types.^17^^,^^18^^,^^19^ Co-IP assays confirmed an interaction between IRX1 and NME3. Notably, the protein abundance of NME3 was not altered upon *IRX1* overexpression or knockdown. Consistent with this, dual-luciferase reporter assays showed that NME3 overexpression alone did not affect *ACLY* promoter activity. This finding supports a model wherein NME3 does not act alone but is recruited by *IRX1* to cooperatively repress *ACLY* transcription. This *IRX1*-*NME3*/*ACLY* interaction thus represents a mechanism of metabolic reprogramming that regulates lipid metabolism in tumor cells.

*ACLY* is a key metabolic enzyme that catalyzes the cytosolic conversion of citrate to acetyl-CoA and oxaloacetate. This reaction provides the fundamental substrate, acetyl-CoA, for *de novo* lipogenesis and is thus pivotal in linking carbohydrate metabolism to lipid biosynthesis, a pathway frequently dysregulated in cancer and metabolic disorders.^20^^,^^21^ *ACLY* expression and activity are frequently upregulated in diverse malignancies, where it fuels tumor cell proliferation and survival by sustaining the high demand for lipids required for membrane biogenesis and signaling molecules. For instance, inhibition of *ACLY* in cancer cells was shown to disrupt fatty acid chain elongation, leading to toxic accumulation of triglycerides and subsequent impairment of proliferation and induction of apoptosis.^22^ Beyond cancer, *ACLY* regulates cellular differentiation and function in other contexts. For example, in immunometabolism, *ACLY* modulates the differentiation of inducible regulatory T cells by reprogramming cellular energy utilization via fatty acid metabolic pathways.^23^ Similarly, in a model of non-alcoholic fatty liver disease (NAFLD), pharmacological inhibition of *ACLY* was demonstrated to reduce hepatic steatosis and concurrently improve mitochondrial function.^24^ Returning to oncogenesis, *ACLY*’s role intersects with critical oncogenic signaling pathways. In prostate cancer, a feedforward loop exists between *ACLY* and the androgen receptor, highlighting *ACLY*’s integral role in sustaining oncogenic metabolism.^25^ Moreover, *ACLY* activity influences the saturation state of newly synthesized fatty acids, a factor that can modify membrane fluidity and the composition of lipid rafts, thereby exerting broader effects on signal transduction and the tumor microenvironment.^26^ Collectively, by driving *de novo* lipogenesis and modulating broader lipid metabolism, *ACLY* is a central effector of the metabolic reprogramming characteristic of cancer cells, underscoring its rationale as a therapeutic target.^27^^,^^28^^,^^29^ The regulatory scope of *ACLY* extends further, as evidenced in viral infections where phosphorylation-activated *ACLY* facilitates neutral lipid synthesis for lipid droplet biogenesis, illustrating the enzyme’s versatile role in diverse metabolic adaptations. In summary, *ACLY* is a multifunctional metabolic nexus that integrates nutritional status with biosynthetic output to regulate not only *de novo* lipogenesis but also cellular energy homeostasis and proliferation. This multifaceted involvement in disease pathophysiology, particularly in cancer, solidifies its therapeutic relevance.

Dysregulated lipid metabolism in tumor cells impacts not only fatty acid synthesis and oxidation but also critically drives cellular proliferation, invasion, and metastasis.^30^^,^^31^ *De novo* fatty acid synthesis, a cornerstone of lipid metabolism, involves a complex network of enzymes and pathways. *ACLY* catalyzes the conversion of citrate to acetyl-CoA, providing the essential two-carbon units for *de novo* fatty acid synthesis, which in turn supplies lipids for membrane biogenesis and energy storage. In this study, we demonstrate that the transcription factor *IRX1* binds to site 1 in the *ACLY* promoter and represses its transcription, thereby attenuating *de novo* fatty acid synthesis in cervical cancer cells. We acknowledge that *IRX1* may regulate additional pathways beyond *ACLY*-mediated lipid metabolism. However, the present study specifically focused on lipid metabolism, which we validated using multiple functional assays. Our data establish *ACLY*-mediated lipid synthesis as a key downstream mechanism of *IRX1*; other potential pathways, however, warrant further investigation.

In summary, our findings identify *IRX1* as a tumor suppressor in cervical cancer, whose expression is epigenetically silenced by promoter CpG island methylation. Mechanistically, NME3 is recruited by *IRX1* to form a transcriptional complex that co-represses *ACLY* expression. This suppression of *ACLY* inhibits *de novo* fatty acid synthesis, thereby impeding cervical cancer progression. In summary, our work delineates the *IRX1*-*NME3*/*ACLY* axis as a key regulatory node in cervical cancer.

### Limitations of the study

Our findings suggest that this axis may be worthy of further investigation. However, this study has several limitations that should be acknowledged. Our functional experiments mainly used *in vitro* overexpression and knockdown systems in cervical cancer cell lines, which may not fully replicate the complex *in vivo* tumor microenvironment. The xenograft model had a relatively small sample size (*n* = 5 per group), and independent validation in a clinical cohort is currently lacking. Additionally, although we identified *ACLY* as a key downstream target of *IRX1*, we did not perform global metabolomic profiling to assess broader metabolic reprogramming. Finally, the precise molecular mechanism by which NME3 facilitates *IRX1*-mediated transcriptional repression of *ACLY* remains to be further investigated.

## Resource availability

### Lead contact

Further information and requests for resources and reagents should be directed to and will be fulfilled by the lead contact, Ke Wang (wangke2@tjmuch.com).

### Materials availability

All materials are available upon reasonable request.

### Data and code availability

• All data reported in this paper will be shared by the lead contact upon request.
• This paper does not report original code.
• Any additional information required to reanalyze the data reported in this paper is available from the lead contact upon request.

## Acknowledgments

This study was supported by funds from the 10.13039/501100001809National Natural Science Foundation of China (grant no. 82202863).

## Author contributions

L.X.: data curation and writing—original draft; H.X.: validation, writing-review and editing, and methodology; Z.Y.: validation, formal analysis, and resources; S.J.: data curation; C.X.: conceptualization and supervision; Y.Y.: conceptualization, supervision, and writing—original draft; W.K.: conceptualization, supervision, and funding acquisition.

## Declaration of interests

The authors declare that they have no competing interests.

## STAR★Methods

### Key resources table

**Table:** 

REAGENT or RESOURCE	SOURCE	IDENTIFIER
**Antibodies**		

Rabbit Polyclonal anti-IRX1	Immunoway	Cat#YT2412;
Rabbit Monoclonal anti-NME3	abcam	Cat#EPR13117;
Rabbit Monoclonal anti-ACLY	Immunoway	Cat#YM8372;
Mouse Monoclonal anti-FANS	proteintech	Cat#66591-1-Ig; RRID:AB_2881951
Rabbit Polyclonal anti-ACSL3	Immunoway	Cat#YN0828;
Rabbit Polyclonal anti-SREBP1	Immunoway	Cat#YT6055;
Rabbit Polyclonal anti-BAX	proteintech	Cat#50599-2-Ig; RRID:AB_2061561
Rabbit Polyclonal anti-GAPDH	proteintech	Cat#10494-1-AP; RRID:AB_2263076
Rabbit Polyclonal anti-Ki-67	proteintech	Cat#27309-1-AP; RRID:AB_2756525
Rabbit Polyclonal anti-HDAC1	Cell Signaling Technology	Cat#2062; RRID:AB_2118523
Rabbit Polyclonal anti-FLAG	proteintech	Cat#80801-2RR; RRID:AB_3670488
Rabbit Polyclonal anti-HA	proteintech	Cat#51064-2-AP; RRID:AB_11042321
Rabbit Anti- IgG/HRP	ZSGB	Cat#ZB-2301; RRID:AB_2747412
Mouse Anti- IgG/HRP	ZSGB	Cat#ZB-2305; RRID:AB_2747415

**Bacterial and virus strains**		

Trans1-T1 phage Resistant Chemically Competent cell	TRANS	Cat#CD501

**Biological samples**		

Xenograft tumor tissues	This paper	N/A

**Chemicals, peptides, and recombinant proteins**		

polybrene	Solarbio	Cat#H8761
puromycin	Solarbio	P8230; CAS:58-58-2
PMSF	Solarbio	IP0280; CAS:329-98-6
RIPA	Solarbio	Cat#R0020
TBS	Servicebio	Cat#G0001-2L
Tween 20	Solarbio	Cat#T8220
PBS	Servicebio	Cat#G0002-2L
DAPI	Solarbio	ID22502; CAS:21718-90-3
4% paraformaldehyde	Solarbio	Cat#P1110
Crystal violet	Solarbio	Cat#C8470
CCK-8 reagent	Beyotime	Cat#C0037
Pierce IP Lysis Buffer	Thermo Fisher Scientific	Cat#87787
sodium citrate buffer	Solarbio	Cat#C1032
3,3′-diaminobenzidine (DAB)	ZSGB	Cat#ZLI-9017
Hematoxylin	Servicebio	Cat#G1004
Ethanol	Bocheng Chemical	NA
Xylene	Bocheng Chemical	NA

**Critical commercial assays**		

Lenti-Pac HIV Expression Packaging Kit	GeneCopoeia	Cat#LT002
Rapid cellular RNA extraction kit	Sparkjade	Cat#AC0205-B
One-Step gDNA Removal and cDNA Synthesis SuperMix kit	TransGen	Cat#AT311-02
Perfectstart Green qPCR SuperMix kit	TransGen	Cat#AQ601-01-V2
BeyoClick™ EdU Cell Proliferation Kit	Beyotime	Cat#C0078S
Tissue/Cell DNA Kit	TIANGEN	Cat#DP602-D
EpiTect bisulfite kit	Beyotime	Cat#59104
EasyPure Quick Gel Extraction Kit	TransGen	Cat#EG101-02
ChIP Assay kit	Beyotime	Cat#P2080S
Hieff Trans universal Transfection Reagent	YESEN	Cat#40808ES03
Triglyceride Assay kits	Beyotime	Cat#S0219S
Cholesterol Assay kits	Beyotime	Cat#S0211S
A-CoA(Acetyl Coenzyme A) ELISA kit	Elabscience	Cat#E-EL-0125
Malonyl-CoA ELISA kit	N/A	N/A

**Experimental models: Cell lines**		

End1/E6E7	ATCC	Cat#CRL-2615
HeLa	Cellverse	Cat#iCell-h088
C33A	Cellverse	Cat#iCell-h031
SiHa	Cellverse	Cat#iCell-h188
CaSki	Cellverse	Cat#iCell-h040
293T	Cellverse	Cat#iCell-h237

**Experimental models: Organisms/strains**		

NSG mice	N/A	N/A

**Oligonucleotides**		

shRNA targeting sequence, IRX1: See Table S1	This paper; singke Biotech	N/A
siRNA targeting sequence, ACLY: See Table S1	Tsingke Biotech	N/A
siRNA targeting sequence, NME3: See Table S1	Tsingke Biotech	N/A
Primer for RT-qPCR, See Table S2	This paper; singke Biotech	N/A
Primer for methylation-specific PCR, See Table S3	This paper; singke Biotech	N/A
Primer for CHIP, See Table S4	This paper; singke Biotech	N/A

**Software and algorithms**		

GraphPad Prism	Prism(Graphpad)	https://www.graphpad.com/
ImageJ	NIH	https://imagej.net/ij/
SnapGene	GSL Biotech	https://www.snapgene.com
PyMOL	Schrödinger, LLC	https://www.pymol.org

### Experimental model and study participant details

#### Animals

Four-week male nude mice were purchased from Bei Jing Si Pei Fu. Five-week-old female NSG mice, were randomly assigned to different experimental groups for subcutaneous xenograft models. All animal procedures were approved by the Ethics Committee of the Tianjin Medical University Cancer Hospital (Approval No: CRP-AE-2022121) and performed in accordance with NIH Guide for the Care and Use of Laboratory Animals.

#### Cells

The End1/E6E7 cell line was obtained from the American Type Culture Collection (ATCC); HeLa, C33A, CaSki, 293T and SiHa cells were acquired from Cellverse Bioscience Technology Co., Ltd. HeLa, CaSki, 293T and SiHa were cultured in Dulbecco’s modified Eagle’s Medium supplemented (DMEM) with 10% fetal bovine serum (FBS) and 1% penicillin/streptomycin at 37°C in a humidified incubator with 5% CO_2_. C33A was cultured in Eagle's Minimum Essential Medium (MEM )and End1/E6E7 was cultured in Eagle’s Minimal Essential Medium and keratinocyte serum-free medium(KSFM). Cultured cells were routinely screened for mycoplasma contamination and used within a limited passage number.

### Method details

#### Plasmids and transfection

Small interfering RNAs (siRNAs) targeting *ACLY* and *NME3*, along with their respective negative controls, were synthesized by Tsingke Biotech (Beijing, China). Overexpression plasmids for *IRX1*, *ACLY*, and *NME3* were constructed in-house. A short hairpin RNA (shRNA) targeting *IRX1* was designed and synthesized by Tsingke Biotech. For transient transfection, cells were transfected using HieffTrans Universal Transfection Reagent (Yeasen, China) following the manufacturer's protocol. For stable transduction, lentiviral particles were packaged using the Lenti-Pac HIV Expression Packaging Kit (GeneCopoeia, USA), the harvested viral supernatants were subsequently used for cell transductiont (HeLa: MOI = 5, C33A: MOI = 50, SiHa: MOI = 10, CaSki: MOI = 5). The detailed sequences of all shRNA and siRNA oligonucleotides used in this study are listed in Table S1.

#### RT-qPCR

Total RNA was extracted from cultured cells using Rapid cellular RNA extraction kit (Sparkjade, China). Subsequently, first-strand cDNA was synthesized from 1 μg of total RNA using the EasyScript One-Step gDNA Removal and cDNA Synthesis SuperMix kit (TransGen, China) according to the manufacturer's instructions. Quantitative real-time PCR (qPCR) was performed using the Perfectstart Green qPCR SuperMix kit (TransGen, China). Each 20 μL reaction mixture contained 10 μL of 2×perfectStart Green qPCR SuperMix, 0.4 μL of each primer (0.2 μM), 2 μL of cDNA template, and nuclease-free water to volume. Thermal cycling protocol: 94°C for 30 s, followed by 40 cycles of 94°C for 5 s, 56°C for 15 s, and 72°C for 10 s. GAPDH mRNA levels served as the endogenous control for normalization and quantified via the 2ˆ-ΔΔCt method. The sequences for all qPCR primers are provided in Table S2.

#### Western blot

Following experimental treatments, cells were lysed on ice using RIPA buffer (Solarbio, China) supplemented with PMSF (Solarbio, China) to extract total protein. Protein concentration was determined using a Pierce BCA Protein Assay Kit (Thermo Fisher Scientific) according to the manufacturer's instructions. Equal amounts of protein (10μg) were separated by sodium dodecyl sulfate-polyacrylamide gel electrophoresis (SDS-PAGE) and subsequently transferred onto polyvinylidene difluoride membranes (Millipore, Bedford, MA, USA). Membranes were blocked with 5% non-fat milk dissolved in TBST for 1 hour at room temperature. They were then incubated overnight at 4°C with specific primary antibodies diluted in the TBST (Servicebio, China)(dilution ratio: anti-IRX1 1:1000; anti-NME3 1:2000; anti-ACLY 1:2000; anti-FASN 1:2000; anti-ACSL3 1:1000; anti-SREBP1 1:1000; anti-BAX 1:2000; anti-GAPDH 1:5000; anti-Ki-67 1:5000; anti-HDAC1 1:1000; anti-Flag 1:10000; anti-HA 1:5000; Goat Anti-Rabbit IgG/HRP Conjugate 1:5000; Goat Anti-Mouse IgG/HRP Conjugate 1:5000). Following primary antibody incubation, membranes were washed six times for 5 minutes each with TBST . Subsequently, membranes were incubated with horseradish peroxidase-conjugated secondary antibodies for 1 hour at room temperature. After thorough washing with TBST, protein bands were visualized using an enhanced chemiluminescence substrate and detected with a chemiluminescence imaging system.

#### Immunofluorescence (IF)

Cells were seeded into a 24-well plate containing coverslips at a density of 3×10^4^ cells per well and cultured overnight. The next day, the cells were washed twice with PBS (Servicebio, China) and then fixed with 4% paraformaldehyde at room temperature for 10 minutes. Subsequently, the cells were permeabilized with PBS containing 0.1% Triton X-100 for 10 minutes, followed by blocking with 3% bovine serum albumin (BSA) at room temperature for 60 minutes to reduce non-specific binding. Subsequently, the cells were incubated with primary antibody against the target protein in a humidified chamber overnight at 4°C. On the third day, the coverslips were returned to the 12-well plate with the cell side facing up, washed three times with PBS, and then incubated with the secondary antibody working solution for 1 hour at room temperature in the dark. 4’, 6-diamidino-2-phenylindole (DAPI) (Solarbio, China) reagent was diluted at a ratio of 1:1000, and the nuclei were stained for 15 minutes at room temperature with the cell side of the coverslip facing up. Finally, the coverslips were sealed with anti-fade mounting medium, and images were observed and captured using a fluorescence microscope.

#### Colony formation assay

Cells were seeded into 6-well plates at a density of 1,000 cells per well in 2 mL of complete culture medium. The plates were then incubated under standard culture conditions (37°C, 5% CO_2_) for 10-14 days, allowing colony formation. The medium was refreshed every 3-4 days. Following incubation, cells were washed twice with PBS, fixed with 4% paraformaldehyde (Solarbio, China) for 20 minutes at room temperature, and then washed again with PBS. Colonies were subsequently stained with 0.1% crystal violet (Solarbio, China) for 30 minutes. After staining, plates were gently rinsed with distilled water to remove excess dye, air-dried, and imaged using a digital camera system.

#### CCK-8 assay

Cells were seeded in 96-well plates at a density of 3,000 cells per well in 200 μL of culture medium and allowed to adhere overnight. Starting the next day (Day 1) and for consecutive days thereafter, 100 μL of CCK-8 reagent (Beyotime, China) was added to each well, followed by incubation for 1 hour at 37°C. The absorbance at 450 nm was then measured using a microplate reader.

#### EdU assay

Cells were seeded in 24-well plates at a density of 2 × 10^4^ cells per well. After appropriate treatment periods, EdU was assessed using the BeyoClick EdU Cell Proliferation Kit (Beyotime, China) strictly according to the manufacturer's instructions. The percentage of EdU-positive cells was quantified by the fluorescence microscope.

#### Transwell migration assay

For the migration assay, cells were serum-starved before preparing the cell suspension. 6×10^4^ cells suspended in 200 μL of serum-free medium were seeded into the upper chamber of a Transwell insert (8-μm pore size; BD Biosciences, USA). The lower chamber was filled with 400 μL of complete medium containing 10% FBS as a chemoattractant. After incubation for 5∼24 hours at 37°C, non-migrating cells on the upper surface of the membrane were removed with a cotton swab. Cells that had migrated to the lower surface were fixed with 4% paraformaldehyde, stained with 0.1% crystal violet, and imaged under an optical microscope.

#### Wound healing assay

To exclude the confounding effect of serum-induced proliferation on cell migration, the wound healing assay was performed under serum-free conditions. cells were serum-starved for 12 hours prior to the experiments. Cells were seeded in 6-well plates and cultured until they reached 90–95% confluence. A sterile 10-μL pipette tip was then used to create a straight scratch across the cell monolayer. After scratching, cells were gently washed with PBS to remove debris and then incubated in medium. Images of the scratch wounds were captured at 0 and 24 hours using an inverted microscope. The migration rate was calculated by measuring the change in scratch width between 0 and 24 hours using ImageJ software.

#### Co-immunoprecipitation (Co-IP) and protein identification

Cells were lysed using RIPA Lysis Buffer (Thermo Fisher Scientific, USA) supplemented with protease and phosphatase inhibitors. For immunoprecipitation, cleared cell lysates were incubated overnight at 4°C with specific primary antibodies (2 μl) and 40 μL Protein A/G Magnetic Beads. The next day, the bead-antibody-protein complexes were washed three times with ice-cold PBS buffer. Immunoprecipitated proteins were then eluted by boiling the beads in loading buffer at 95°C for 10 minutes. The eluted proteins were subsequently separated by SDS-PAGE for immunoblotting analysis. For protein identification, proteins immunoprecipitated under identical conditions were separated by SDS-PAGE and stained with Coomassie Brilliant Blue. The resulting peptides were analyzed by liquid chromatography-tandem mass spectrometry. The data acquisition and bioinformatic analysis were performed by Novogene Co., Ltd. (Beijing, China).

#### Immunohistochemistry (IHC)

Following deparaffinization and rehydration, antigen retrieval was performed by heating the tissue sections in 10 mM sodium citrate buffer (Solarbio, China) using a microwave. After cooling to room temperature, endogenous peroxidase activity was quenched by incubating the sections in 3% hydrogen peroxide for 10 minutes. Non-specific binding sites were blocked by incubating the sections with 5% BSA in PBS for 1 hour at room temperature. The sections were then incubated overnight at 4°C with specific primary antibodies diluted in blocking buffer. Unbound primary antibody was removed by washing the sections three times for 5 minutes each with PBST. Subsequently, the sections were incubated with an appropriate horseradish peroxidase -conjugated secondary antibody for 1 hour at room temperature. Following a similar washing step with PBST, immunoreactivity was visualized by applying a 3,3’-diaminobenzidine (DAB) (ZSGB, China) chromogen substrate. Finally, the sections were counterstained with hematoxylin (Servicebio, China), dehydrated through a graded ethanol (Bocheng Chemical, China) series, cleared in xylene (Bocheng Chemical, China), and mounted with a permanent mounting medium. Stained sections were examined and imaged under a light microscope.

#### DNA isolation and bisulfite sequencing PCR (BSP)

Genomic DNA was extracted using the commercial kit (TIANGEN, China) according to the manufacturer’s protocol. Bisulfite conversion was performed on 500 ng of genomic DNA using the EpiTect bisulfite kit (Beyotime, China), wherein unmethylated cytosines are deaminated to uracil, whereas methylated cytosines remain protected. The bisulfite-converted DNA was purified and subsequently used as template for PCR amplification with bisulfite-specific primers. PCR products were purified using the EasyPure Quick Gel Extraction Kit (Transgene, China). Purified amplicons were ligated into the pGEM-T Easy vector (Promega, USA) and transformed into competent cells. Ten randomly selected clones were subjected to Sanger sequencing. Sequencing traces were analyzed using QUMA (Quantification Tool for Methylation Analysis). Methylation levels were quantified as the percentage of unconverted cytosines over the total of cytosines and thymines at each CpG dinucleotide. Bisulfite sequencing primers are listed in Table S3.

#### Chromatin immunoprecipitation (ChIP)-qPCR and luciferase reporter assays

ChIP assays were performed using a commercial ChIP kit (Beyotime, China) according to the manufacturer's protocol. Briefly, cells were cross-linked with 1% formaldehyde for 10 minutes at room temperature. Cells were then lysed, and the chromatin was sheared into fragments of 200–500 bp by sonication. The clarified chromatin lysates were incubated overnight at 4°C with antibodies specific to the target protein. The next day, protein A/G agarose beads were added to capture the antibody-chromatin complexes. The beads were then collected and washed to remove non-specific binding. After the final wash, the protein-DNA cross-links were reversed by incubation at 65°C for 4∼6 hours. The immunoprecipitated DNA was purified and analyzed by qPCR using the primers listed in Table S4.

For dual-luciferase reporter assays, a DNA fragment containing the putative *ACLY* promoter region was cloned upstream of the firefly luciferase gene in the pGL3-Basic vector (Promega, China). Cells were co-transfected with the constructed pGL3-ACLY promoter reporter plasmid, the pRL-TK Renilla luciferase control plasmid (Promega, China), and either an *IRX1* overexpression plasmid or its corresponding empty vector control, using Hieff Trans universal Transfection Reagent (YESEN, China). After 48 hours, luciferase activities were measured using the Dual-Luciferase® Reporter Assay System (TransGen Biotech, China) following the manufacturer’s instructions. Firefly luciferase activity was normalized to Renilla luciferase activity for each sample to control for transfection efficiency.

#### Measurement of triglyceride and cholesterol

Intracellular cholesterol and triglyceride levels in cervical cancer cells were quantified using commercial assay kits (Beyotime, China). Following the manufacturer’s protocol, 50 μL of clarified cell lysate supernatant was mixed with 150 μL of the respective working reagent in a 96-well plate. The reaction mixture was then incubated at 37°C for 60 minutes, protected from light. Following incubation, the absorbance at 570 nm was measured using a microplate reader. The concentrations of total cholesterol and triglycerides were interpolated from a standard curve run in parallel. The results were normalized to protein concentration.

#### Measurement of Acetyl-CoA and Malonyl-CoA content

The intracellular levels of acetyl-CoA and malonyl-CoA were quantified using commercially available enzyme-linked immunosorbent assay (ELISA) kits (for acetyl-CoA: Elabscience, China; for malonyl-CoA: Enzyme Immunity Technology Co., Ltd.) following the respective manufacturers' protocols. Briefly, approximately 1×10^6^ cells were harvested, washed with cold PBS, and lysed in 200 μL of appropriate lysis buffer provided with the kits. The lysates were then centrifuged at 12,000 ×g for 15 minutes at 4°C to obtain clarified supernatants. Subsequent assay steps were performed strictly in accordance with the detailed instructions of each ELISA kit. Absorbance at 450 nm was measured using a BioTek microplate reader. The concentration of each metabolite was determined by interpolating sample absorbance values against a concurrently run standard curve. Final data were normalized to the total protein concentration of the corresponding lysate, as determined by a bicinchoninic acid protein assay. The results were normalized to protein concentration.

#### Oil Red O staining

To assess lipid deposition, cells were subjected to Oil Red O staining. Briefly, cells were washed twice with PBS to remove the culture medium. Subsequently, cells were fixed with 4% paraformaldehyde in PBS for 10 minutes at room temperature. After fixation, cells were washed three times with PBS. To reduce non-specific background, cells were incubated with 200 μL of the provided staining wash buffer (Beyotime, China) for 10 minutes at room temperature. Cells were then stained with a freshly prepared Oil Red O working solution for 15 minutes at room temperature, protected from light. Following Oil Red O staining, cells were washed with distilled water and were imaged using an inverted light microscope (Nikon, Japan).

#### Xenograft

Cells from the *IRX1*-overexpressing group, *ACLY*-overexpressing group and the control group were resuspended in a mixture of PBS and growth factor-reduced Matrigel (5% final concentration; BD Biosciences, USA). The cell-Matrigel mixture was then subcutaneously injected into the right flank of each mouse. Four-week-old NSG mice were randomly allocated into the experimental and control groups (*n* =5 per group). Tumor volume was measured and recorded weekly. Animal health and behavior were monitored daily. Mice were euthanized when tumors reached a maximum diameter of 1.0 cm, in strict compliance with the Institutional Animal Care and Use Committee (IACUC) protocol. Upon euthanasia, tumors were immediately excised, and photographed. All animal experiments were approved by the Animal Ethics Committee.

### Quantification and statistical analysis

Statistical analyses were performed using GraphPad Prism software. Comparisons between two groups were performed using an unpaired Student's t-test. For comparisons among three or more groups, one-way ANOVA with Tukey's post hoc test was applied. Data are presented as the mean ± standard deviation (SD). Differences with a *p*-value less than 0.05 were considered statistically significant.Data are derived from at least three independent experiments.Results are expressed as follows: ∗*p* < 0.05, ∗∗*p* < 0.01, ∗∗∗*p* < 0.001, and ns denotes no significant difference.

### Additional resources

All other items: any additional information required to reanalyze the data reported in this paper is available from the lead contact upon request.

## References

[1] Phianmongkhol Y., Suwan N., Srisomboon J., Kietpeerakool C. (2011). Knowledge about human papillomavirus infection and cervical cancer prevention among nurses in Chiang Mai University Hospital, Thailand. Asian Pac. J. Cancer Prev. APJCP.

[2] Nakabayashi N., Hirose M., Suzuki R., Suzumiya J., Igawa M. (2018). How asymptomatic are early cancer patients of five organs based on registry data in Japan. Int. J. Clin. Oncol..

[3] Chen Y., Liu S., Yin X. (2025). Progress and prospects of the combination of BMI1-targeted therapy and immunotherapy in cervical cancer. Am. J. Cancer Res..

[4] Wargantiwar S., Bhattacharya S., Kanugo A. (2025). Surgical Advancements, Immunotherapy, Targeted and Conventional Therapies, Biopsy, Colposcopy, and Pap Smear Integration in the Management of Cervical Cancer. Curr. Med. Chem..

[5] Malagón T., Drolet M., Boily M.C., Laprise J.F., Brisson M. (2015). Changing inequalities in cervical cancer: modeling the impact of vaccine uptake, vaccine herd effects, and cervical cancer screening in the post-vaccination era. Cancer Epidemiol. Biomarkers Prev..

[6] Chigbu C.O., Onyebuchi A.K., Onyeka T.C., Odugu B.U., Dim C.C. (2017). The impact of community health educators on uptake of cervical and breast cancer prevention services in Nigeria. Int. J. Gynaecol. Obstet..

[7] Jung I.H., Jung D.E., Chung Y.Y., Kim K.S., Park S.W. (2019). Iroquois Homeobox 1 Acts as a True Tumor Suppressor in Multiple Organs by Regulating Cell Cycle Progression. Neoplasia.

[8] Demokan S., Chuang A.Y., Pattani K.M., Sidransky D., Koch W., Califano J.A. (2014). Validation of nucleolar protein 4 as a novel methylated tumor suppressor gene in head and neck cancer. Oncol. Rep..

[9] Liu X., Zhang J., Liu L., Jiang Y., Ji J., Yan R., Zhu Z., Yu Y. (2018). Protein arginine methyltransferase 5-mediated epigenetic silencing of IRX1 contributes to tumorigenicity and metastasis of gastric cancer. Biochim. Biophys. Acta Mol. Basis Dis..

[10] Guo X., Liu W., Pan Y., Ni P., Ji J., Guo L., Zhang J., Wu J., Jiang J., Chen X. (2010). Homeobox gene IRX1 is a tumor suppressor gene in gastric carcinoma. Oncogene.

[11] Küster M.M., Schneider M.A., Richter A.M., Richtmann S., Winter H., Kriegsmann M., Pullamsetti S.S., Stiewe T., Savai R., Muley T., Dammann R.H. (2020). Epigenetic Inactivation of the Tumor Suppressor IRX1 Occurs Frequently in Lung Adenocarcinoma and Its Silencing Is Associated with Impaired Prognosis. Cancers.

[12] Bennett K.L., Karpenko M., Lin M.T., Claus R., Arab K., Dyckhoff G., Plinkert P., Herpel E., Smiraglia D., Plass C. (2008). Frequently methylated tumor suppressor genes in head and neck squamous cell carcinoma. Cancer Res..

[13] Mares J., Sedlácek Z. (2000). DNA methylation and neoplasms. Cas. Lek. Ces..

[14] Li W., Chen B. (2013). Aberrant DNA methylation in human cancers. J Huazhong Univ Sci Technolog. Med. Sci..

[15] Paluszczak J., Baer-Dubowska W. (2006). W. Epigenetic diagnostics of cancer--the application of DNA methylation markers. J. Appl. Genet..

[16] Hartsough M.T., Steeg P.S. (2000). Nm23/nucleoside diphosphate kinase in human cancers. J. Bioenerg. Biomembr..

[17] Min S.H., Zheng Q.Q. (2021). Clinicopathological and prognostic significance of NM23 expression in patients with non-small cell lung cancer: A systematic review and meta-analysis. Medicine (Baltim.).

[18] Wu H., Huang X., Chen S., Li S., Feng J., Zouxu X., Xie Z., Xie X., Wang X. (2020). Comprehensive analysis of the NME gene family functions in breast cancer. Transl. Cancer Res..

[19] Kim S., Kang M., Jeong S., Kim J., Kim K.O., Lee W.S., Baek J.H., Kim J.H., Nam S. (2025). Elucidating prognostic significance of purine metabolism in colorectal cancer through integrating data from transcriptomic, immunohistochemical, and single-cell RNA sequencing analysis. Mol. Oncol..

[20] Ke X., Zhang R., Li P., Zuo L., Wang M., Yang J., Wang J. (2022). Hydrochloride Berberine ameliorates alcohol-induced liver injury by regulating inflammation and lipid metabolism. Biochem. Biophys. Res. Commun..

[21] Shan J., Li X., Sun R., Yao Y., Sun Y., Kuang Q., Dai X., Sun Y. (2024). Palmitoyltransferase ZDHHC6 promotes colon tumorigenesis by targeting PPARγ-driven lipid biosynthesis via regulating lipidome metabolic reprogramming. J. Exp. Clin. Cancer Res..

[22] Migita T., Okabe S., Ikeda K., Igarashi S., Sugawara S., Tomida A., Soga T., Taguchi R., Seimiya H. (2014). Inhibition of ATP citrate lyase induces triglyceride accumulation with altered fatty acid composition in cancer cells. Int. J. Cancer.

[23] Tian M., Hao F., Jin X., Sun X., Jiang Y., Wang Y., Li D., Chang T., Zou Y., Peng P. (2021). ACLY ubiquitination by CUL3-KLHL25 induces the reprogramming of fatty acid metabolism to facilitate iTreg differentiation. eLife.

[24] Zhang M., Ji J., Lei Y., Qin F., Tao Y., Li N., Bian J., Li Z., Lai M., Qiu Z. (2025). Dual inhibition of hepatic ACLY and ACSS2: A synergistic approach to combat NAFLD through lipogenesis reduction and mitochondrial enhancement. Pharmacol. Res..

[25] Shah S., Carriveau W.J., Li J., Campbell S.L., Kopinski P.K., Lim H.W., Daurio N., Trefely S., Won K.J., Wallace D.C. (2016). Targeting ACLY sensitizes castration-resistant prostate cancer cells to AR antagonism by impinging on an ACLY-AMPK-AR feedback mechanism. Oncotarget.

[26] Yan Y., Zhou Y., Li J., Zheng Z., Hu Y., Li L., Wu W. (2021). Sulforaphane downregulated fatty acid synthase and inhibited microtubule-mediated mitophagy leading to apoptosis. Cell Death Dis..

[27] Sur S., Nakanishi H., Flaveny C., Ippolito J.E., McHowat J., Ford D.A., Ray R.B. (2019). Inhibition of the key metabolic pathways, glycolysis and lipogenesis, of oral cancer by bitter melon extract. Cell Commun. Signal..

[28] Xu Y., Zhang Z., Xu D., Yang X., Zhou L., Zhu Y. (2021). Identification and integrative analysis of ACLY and related gene panels associated with immune microenvironment reveal prognostic significance in hepatocellular carcinoma. Cancer Cell Int..

[29] Bandyopadhyay D., Tran E.T., Patel R.A., Luetzen M.A., Cho K., Shriver L.P., Patti G.J., Varvares M.A., Ford D.A., McCommis K.S. (2024). Momordicine-I suppresses head and neck cancer growth by modulating key metabolic pathways. Cell Commun. Signal..

[30] Irshad R., Tabassum S., Husain M. (2023). Aberrant Lipid Metabolism in Cancer: Current Status and Emerging Therapeutic Perspectives. Curr. Top. Med. Chem..

[31] Corbet C., Feron O. (2017). Emerging roles of lipid metabolism in cancer progression. Curr. Opin. Clin. Nutr. Metab. Care.

