# Supplementary material for: IRX1 suppresses cervical squamous cell carcinoma progression by downregulating ACLY expression to inhibit lipid synthesis
Source: iScience. 2026 Sep 1;29(9):117115. doi: 10.1016/j.isci.2026.117115 (PMC13562399; doi:10.1016/j.isci.2026.117115)
Supplement: Supplementary file 1 [file mmc1.pdf]

iScience, Volume *29*

Supplemental information

IRX1 suppresses cervical squamous cell carcinoma progression by downregulating *ACLY* expression to inhibit lipid synthesis

Xiangyu Liu, Xinwei Hou, Yuanyuan Zhang, Jindong Sheng, Yue Yu, Xuchen Cao, and Ke Wang

Figure S1. *IRX1* inhibits CESC proliferation and migration *in vitro*, related to Figure 1.
(A) The IRX1 expression in *IRX1*-knockdown CasKi cells (left) or *IRX1*-overexpressing C33A cells (right), as well as control cells by western blot. **(B-D)** The effect of *IRX1* on CESC proliferation was determined by CCK-8 (B), EdU (C) and colony formation (D) assays. Scale bar, 250 μm. (D). **(E and F)** The effect of *IRX1* on CESC migration and invasion was determined by Transwell (E) and wound healing (F) assays. Scale bar, 100 μm. Data are presented as mean ± SD from three independent biological replicates (n = 3). *p < 0.05, **p < 0.01, ***p < 0.001 by unpaired two-tailed t-test (B (right), C (down), D (right), E(down), F(down)) and one-way ANOVA followed by Tukey's post hoc test (B (left), C (up), D(left), E(up), F(up)).

Figure S1

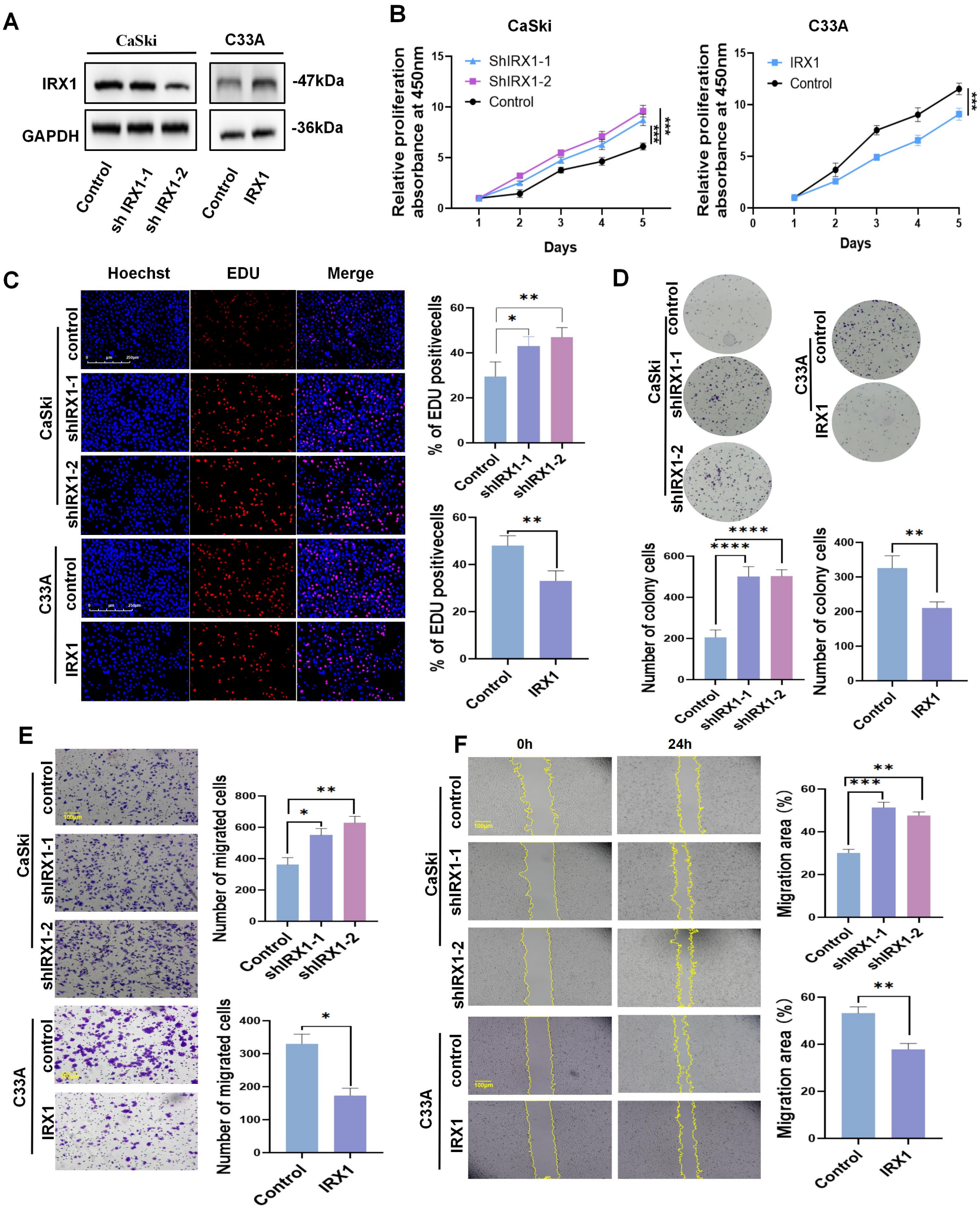

Figure S2. ***IRX1*** **regulates fatty acid synthesis, related to Figure 3 and Figure 4.**

(A) The IRX1 expression in lipid metabolism-related genes (ACLY, FANS, SREBP1, ACSL3) in *IRX1*-overexpressing C33A (left) or *IRX1*-knockdown CasKi cells (right), as well as control cells by Western blot. **(B)** The lipid droplets abundance in cells as in (A) detected by Oil Red O staining. Scale bar, 100 μm. **(C-F)** The measurement of triglyceride (C), cholesterol (D), acetyl-CoA(E), and malonyl-CoA (F) contents in cells as in (A). **(G-K)** The measurement of triglyceride (G), cholesterol (H), Acetyl-CoA (I) and malonyl-CoA (K) contents in *IRX1*/*ACLY*-overexpressing C33A cells or *IRX1*/*ACLY*-knockdown CasKi cells, as well as control cells by ELISA. Data are presented as mean ± SD from three independent biological replicates (n = 3). *$p < 0.05$, **$p < 0.01$, ***$p < 0.001$ by unpaired two-tailed t-test (C (left), D (left), E (left), F(left)) and one-way ANOVA followed by Tukey's post hoc test (C (right), D (right), E(right), F(right), G, H, I, K).

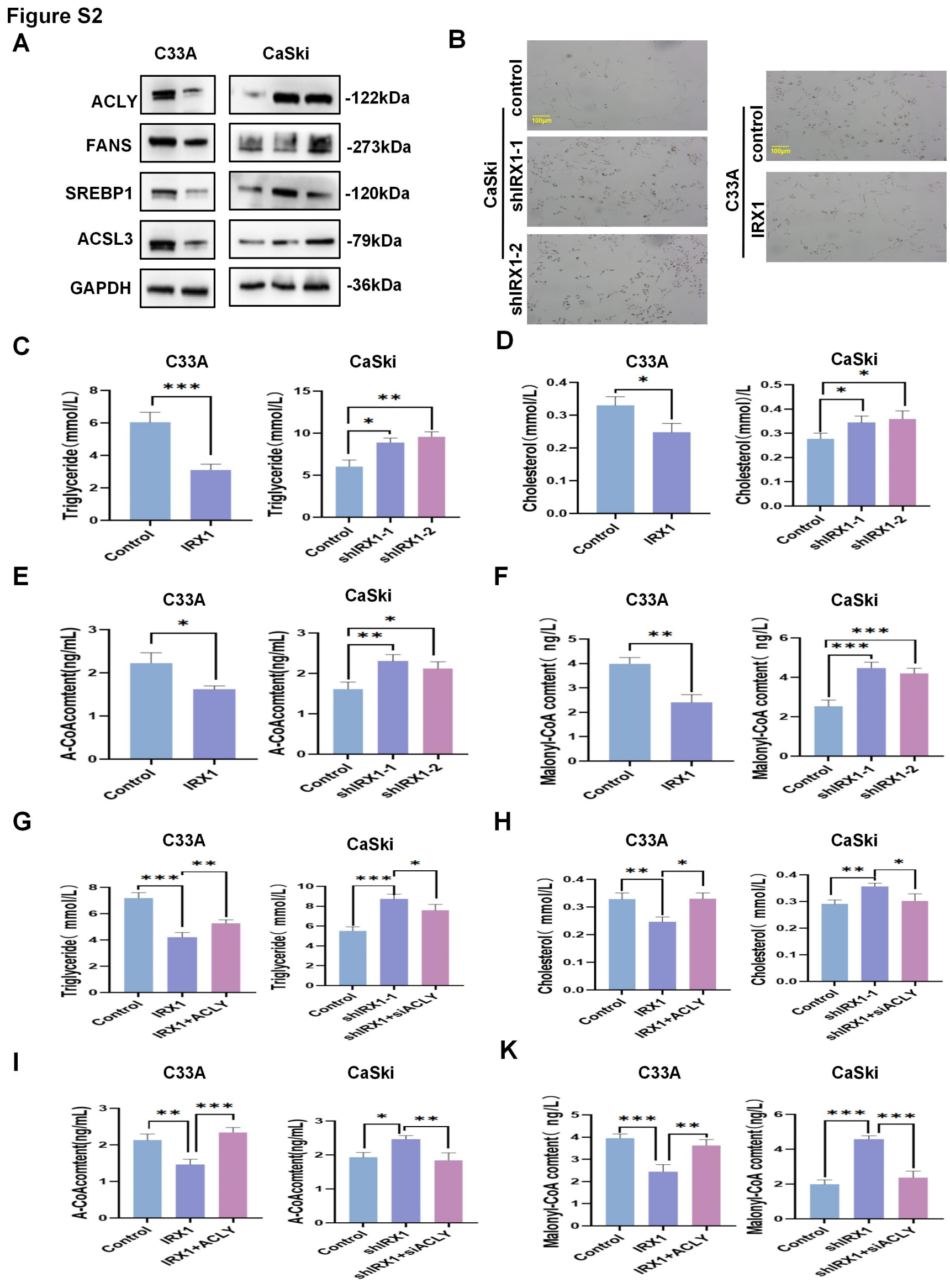

Table S1. Sequence for shRNA and Oligonucleotides of siRNA, related to STAR Methods.

shIRX1-1-F	CCGGGATGGGCTCGCAGTATGAACTCTCGAGAGTTCATACTGCGAGCCCATCTTTTTG
shIRX1-1-R	AATTCAAAAAGATGGGCTCGCAGTATGAACTCTCGAGAGTTCATACTGCGAGCCCATC
shIRX1-2-F	CCGGGCAAGTTCTCCAACTGGACCACTCGAGTGGTCCAGTTGGAGAACTTGCTTTTTG
shIRX1-2-R	AATTCAAAAAGCAAGTTCTCCAACTGGACCACTCGAGTGGTCCAGTTGGAGAACTTGC
siACLY-F	CGAGAAAGCUGAUCAAGAAGG
siACLY-R	UUCUUGAUCAGCUUUCUCGUG
siNME3-F	GGCCGCCUUGUCAAGUAUAUG
siNME3-R	UAUACUUGACAAGGCGGCCGU

Table S2. Sequence for RT-Qpcr (5' to 3'), related to STAR Methods.

IRX1-F	TCTCAGCCTCTTCTCGCAGATG
IRX1-R	GGTCCCCGTATTGGAACTGGC
NME3-F	CATCTTCGCTAACCTCTTCCCC
NME3-R	CCCGGAGGCCATATACTTGAC
GAPDH-F	GGAGCGAGATCCCTCCAAAAT
GAPDH-R	GGCTGTTGTCATACTTCTCATGG

Table S3. Sequence for methylation-specific PCR (5' to 3'), related to STAR Methods.

BSP -F	GCTTTCTGGGTGAAGAGAAAGC
BSP -R	CTCCAGGGGACATCTTTGGCAGAA

Table S4. Sequence for CHIP (5' to 3'), related to STAR Methods.

Site 1-F	GTGAATAATAGAAACAACAGGGTCC
Site 1 -R	CCCCTGCCCTCACCTTTTG
Site 2-F	CATATGTTGCTTACTGGCCACCTT
Site 2 -R	CCCATCATTTGCTGGCAGATTGG
Site 3-F	CAGAACGAGATCCTGTCTCTG
Site 3 -R	CTGAGGTGCTAACCACAAAACTA
